# An optimized transfer learning-based approach for automatic diagnosis of COVID-19 from chest x-ray images

**DOI:** 10.7717/peerj-cs.555

**Published:** 2021-05-27

**Authors:** Waleed M. Bahgat, Hossam Magdy Balaha, Yousry AbdulAzeem, Mahmoud M. Badawy

**Affiliations:** 1 Information Technology Department, Faculty of Computer and Information, Mansoura University, Mansoura, Egypt; 2 Computers and Systems Engineering Department, Faculty of Engineering, Mansoura University, Mansoura, Egypt; 3 Computer Engineering Department, Misr Higher Institute for Engineering and Technology, Mansoura, Egypt

**Keywords:** Classification, COVID-19, Deep convolutional neural network, Transfer learning, X-ray images

## Abstract

Accurate and fast detection of COVID-19 patients is crucial to control this pandemic. Due to the scarcity of COVID-19 testing kits, especially in developing countries, there is a crucial need to rely on alternative diagnosis methods. Deep learning architectures built on image modalities can speed up the COVID-19 pneumonia classification from other types of pneumonia. The transfer learning approach is better suited to automatically detect COVID-19 cases due to the limited availability of medical images. This paper introduces an Optimized Transfer Learning-based Approach for Automatic Detection of COVID-19 (OTLD-COVID-19) that applies an optimization algorithm to twelve CNN architectures to diagnose COVID-19 cases using chest x-ray images. The OTLD-COVID-19 approach adapts Manta-Ray Foraging Optimization (MRFO) algorithm to optimize the network hyperparameters’ values of the CNN architectures to improve their classification performance. The proposed dataset is collected from eight different public datasets to classify 4-class cases (COVID-19, pneumonia bacterial, pneumonia viral, and normal). The experimental result showed that DenseNet121 optimized architecture achieves the best performance. The evaluation results based on Loss, Accuracy, F1-score, Precision, Recall, Specificity, AUC, Sensitivity, IoU, and Dice values reached 0.0523, 98.47%, 0.9849, 98.50%, 98.47%, 99.50%, 0.9983, 0.9847, 0.9860, and 0.9879 respectively.

## Introduction

Coronavirus Disease 2019 (COVID-19) had been detected in Wuhan, China, at the end of the year 2019 and represented a severe health issue worldwide. The recent Coronavirus (COVID-19) has been declared a pandemic by the World Health Organization (WHO) in March 2020. Mankind faces many pandemics like Spanish flu in 1918, Severe Acute Respiratory Syndrome (SARS) in 2003, and presently COVID-19. These infections are airborne and might, therefore, promptly transmittable taint expansive bunches of people (Ghayvat et al., 2021). There is no crisis within the history of mankind that comes anyplace close to the scale of the COVID-19 widespread, with such numerous nations being influenced at the identical time in a very brief time (World Health Organization, 2020a). The infodemic that the widespread of such a scale has caused makes it troublesome for people to get solid direction around COVID-19 avoidance and remedy (Choudrie et al., 2021).

**Figure 1:**
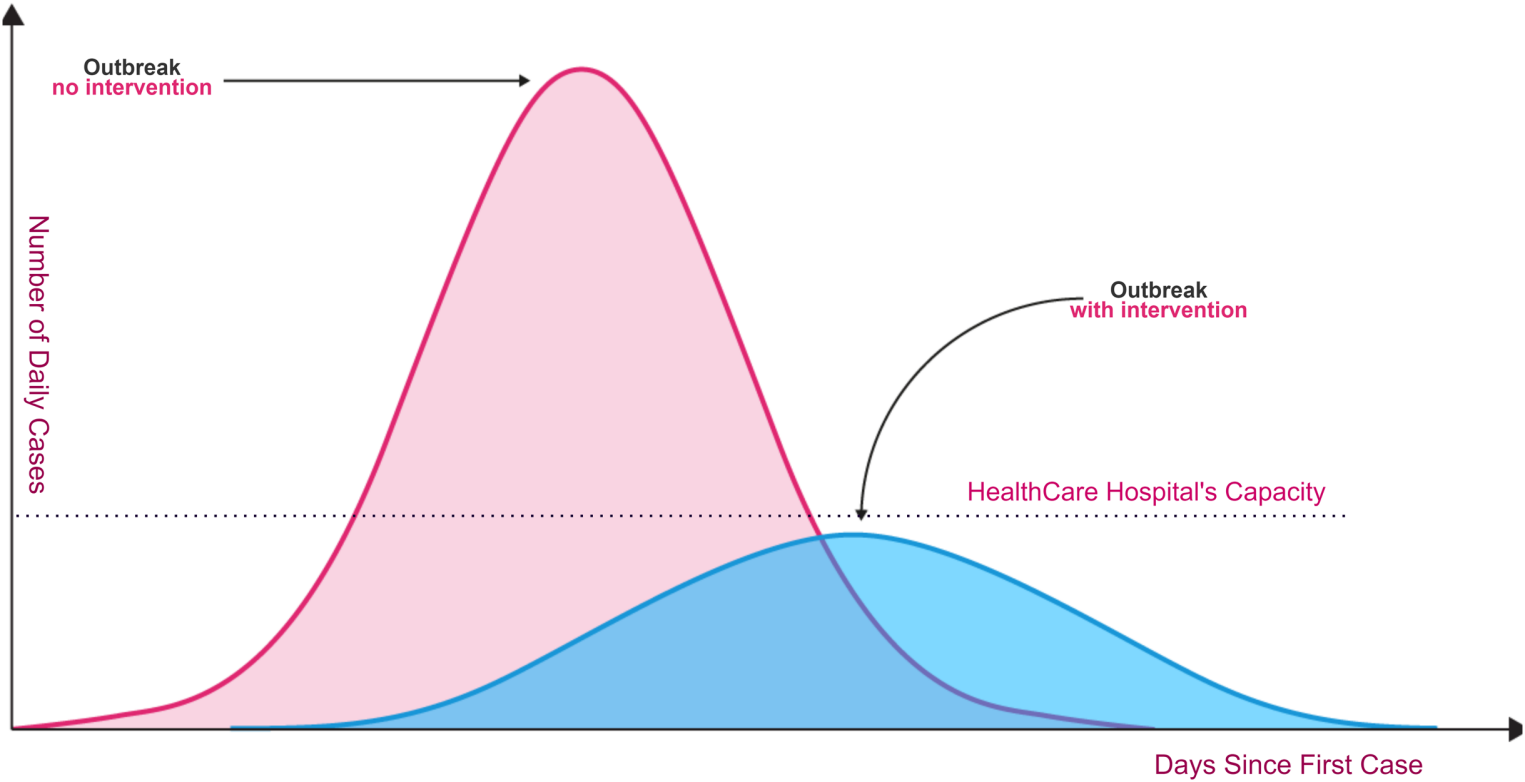
Coronavirus outbreaks and lowering infection rates (World Health Organization, 2020b).

Unfortunately, the spread of COVID-19 is exponentially, and the transmission process is not clearly understood. Today, dealing with Coronavirus is an important healthcare challenge for humanity all over the world. Even though the Coronavirus infection in common incorporates small or no side effects, it causes lethal pneumonia in 2:8% of the infected patients (Hoehl et al., 2020; Lu et al., 2020; Sohrabi et al., 2020). Normally it takes 5:6 days from the disease day with the infection for indications to appear. However, it may take up to 14 days in some cases. As shown in Fig. 1, without rapid testing, the number of COVID-19 cases could increase beyond the healthcare system’s capacity to deal with serious cases (red curve in relation to healthcare system capacity line). Fast and accurate detection reduces the daily rate of new cases (flattening the blue curve).

**Figure 2:**
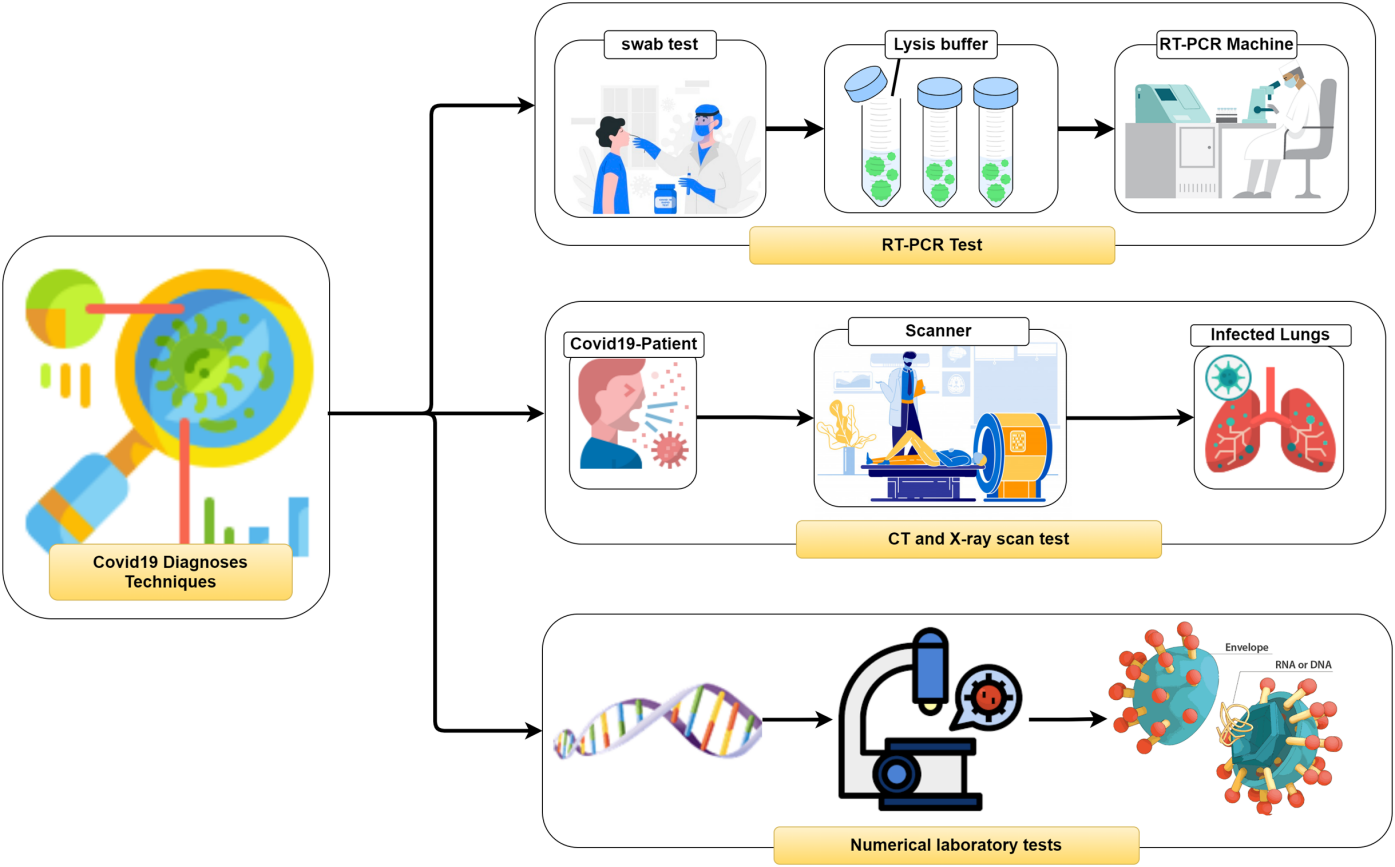
Coronavirus diagnose techniques.

It is vital to distinguish (i.e., diagnose) the COVID-19 as quickly as conceivable and in an exact way to diminish the fast spread of the infection. In general, three different methodologies, as seen in Fig. 2, can be used to recognize COVID-19 patients as follows: (1) RealTime reverse transcriptase-polymerase Chain Reaction (RT-PCR), (2) Chest Computed Tomography (CT) imaging scan, and (3) Numerical laboratory tests. The current common strategy to recognize COVID-19 patients is the RT-PCR test (Ai et al., 2020) which is fairly sensitive and reliable. Unfortunately, depending only on the RT-PCR causes a remarkable delay in the diagnosis of the suspected patients. Besides, utilizing the RT-PCR test has numerous troubles within the extremely hit regions, particularly amid early flare-ups of this disease. On the other hand, the high false-negative rates are a true challenge for the test labs. Many factors affect the sample result, such as test planning and quality control (Liang, 2020).

As a result, chest imaging, such as chest CT or chest X-ray (CXR), is utilized as a first-line examination to detect the virus infection (Rubin et al., 2020; Wong et al., 2020). Chest imaging technologies, especially CXR, are broadly accessible and economical. For this reason, radiologists use chest imaging to detect early lesions in the lung at high speed and sensitivity. Concerning the COVID-19, several aspects of lung anomaly such as bilateral abnormalities, interstitial abnormalities, lung consolidation, and ground-glass opacities are showed in chest images (Guan et al., 2020). Consequently, examining the suspected patients’ chest images presents an essential part remarkable potential for screening processes, and early determination of COVID-19 disease (Kandaswamy et al., 2014). Unfortunately, the diagnosis process mainly relies on the radiologists’ visual diagnosis, which leads to many issues. At first, it takes an exceptionally long time to diagnose since chest imaging contains hundreds of slices, which takes a long time to analyze. Much other pneumonia has similar aspects to COVID-19, so, only radiologists with accumulative experience to diagnose COVID-19 disease.

The artificial intelligence (AI) branch, especially deep learning (DL), has been used to automatically identify lung diseases in medical imaging with significantly diagnostic accuracy (Guan et al., 2020; Rubin et al., 2020). Deep learning is efficient in dealing with the medical dataset, particularly those datasets containing a huge number of training samples. Recently, many research papers address the detection of COVID-19 pneumonia and classify the severity of COVID-19. These research studies try to automatically detect the infected patients early to help society by isolating them to prevent or decrease the native spread. Deep transfer learning (DTL) is a deep learning approach trained on a source problem and then reused to solve a target problem. DTL can be used for two reasons: (1) the transfer of the extracted features from the source issue (Kandaswamy et al., 2014) and (2) the retrain of only the network unfrozen layers by constraining not to over train for the target mission (Yosinski et al., 2014). Employing Convolution Neural networks (CNN) using Transfer Learning is recently used to achieve very promising approaches to diagnose several diseases such as cancer (Bhatt, Ganatra & Kotecha, 2021) and Parkinson (Suzuki, 2017).

Although the research applied in convolutional neural network architectures achieved promising results to detect the COVID-19, the huge number of existing hyperparameters still represents a challenge to achieve better and promising results (Ng et al., 2020). There are recent investigations to optimize CNN’s hyperparameters by utilizing metaheuristics. The hyperparameters of the CNN network, such as learning rate, kernel size, and kernel type of convolutional blocks, should be tuned to enhance the network’s performance.

Zhao, Zhang & Wang (2020) proposed the Manta Ray Foraging Optimization (MRFO) algorithm, inspired by manta rays’ intelligent behaviors. MRFO overcomes the major drawbacks of well-known metaheuristics algorithms, such as slow search speed and easy premature convergence. MRFO outperforms the other optimization algorithms to search precision, convergence rate, stability, and local optimal value avoidance (Wong et al., 2020). This study suggests using the MRFO algorithm to fine-tune the CNN network’s hyperparameters to achieve more accurate results.

This study’s main objective is to introduce an Optimized Transfer Learning-based Approach for Automatic Detection of COVID-19 (OTLD-COVID-19). The OTLD-COVID-19 approach performs the classification and recognition of the lung X-ray images for COVID-19 diseases. The OTLD-COVID-19 approach uses CNN and MRFO algorithm for the parameters and hyperparameters optimizations, respectively. The rest of the paper is structured as follows: “Background” represents the CNNs and MRFO Algorithm theoretical background. “Related Work” discusses the related work. “The Proposed OTLD-COVID-19 Approach” shows the proposed OTLD-COVID-19 approach details. The results, achieved by the proposed technique, are presented in “Experiments, Results and Discussion”. Finally, the conclusions and future work are drawn.

### Paper contributions

In this paper, an approach for automatically detecting COVID-19 using transfer learning is proposed to achieve diagnosis reliably. The OTLD-COVID19 approach works on lung X-ray images to achieve both recognition and classification of COVID-19 diseases. This approach aims to achieve high performance in both processes. To that end, it seeks the best hyperparameters combination to optimize CNN’s parameters. There are many meta-heuristics optimization algorithms with different approaches, including try-and-error, deterministic, and stochastic. This study uses a meta-heuristic optimizer to search the scope automatically. In this vein, to improve the classification performance, the OTLD-COVID-19 approach utilized CNN and MRFO algorithm for the automatic parameters and hyperparameters optimization respectively. The OTLD-COVID-19 approach is used as a pillar to improve overall accuracy and reduce computational cost. The contributions of the current study are summarized in points as follow:

- Proposing the OTLD-COVID-19 approach for rapid COVID-19 diagnosis using automated X-ray image processing.
- Twelve popular CNN architectures, with four classes (“COVID-19,” “Bacterial Pneumonia,” “Viral Pneumonia,” and “Normal”), are used in the transfer learning technique for the detection of COVID-19 patients.
- The Manta-Ray Foraging Optimization (MRFO) algorithm is used to optimize the network hyperparameters’ values to enhance the classification performance.
- The proposed technique is adaptable and scalable; there is no need to manually assign the CNN architecture’s hyperparameters values.
- Regularization, optimization, dropout, and data augmentation are studied through the different reported experiments.
- The proposed technique is compared with the other state-of-the-art techniques and studies. The achieved results of the standard performance metrics are very promising.

**Figure 3:**
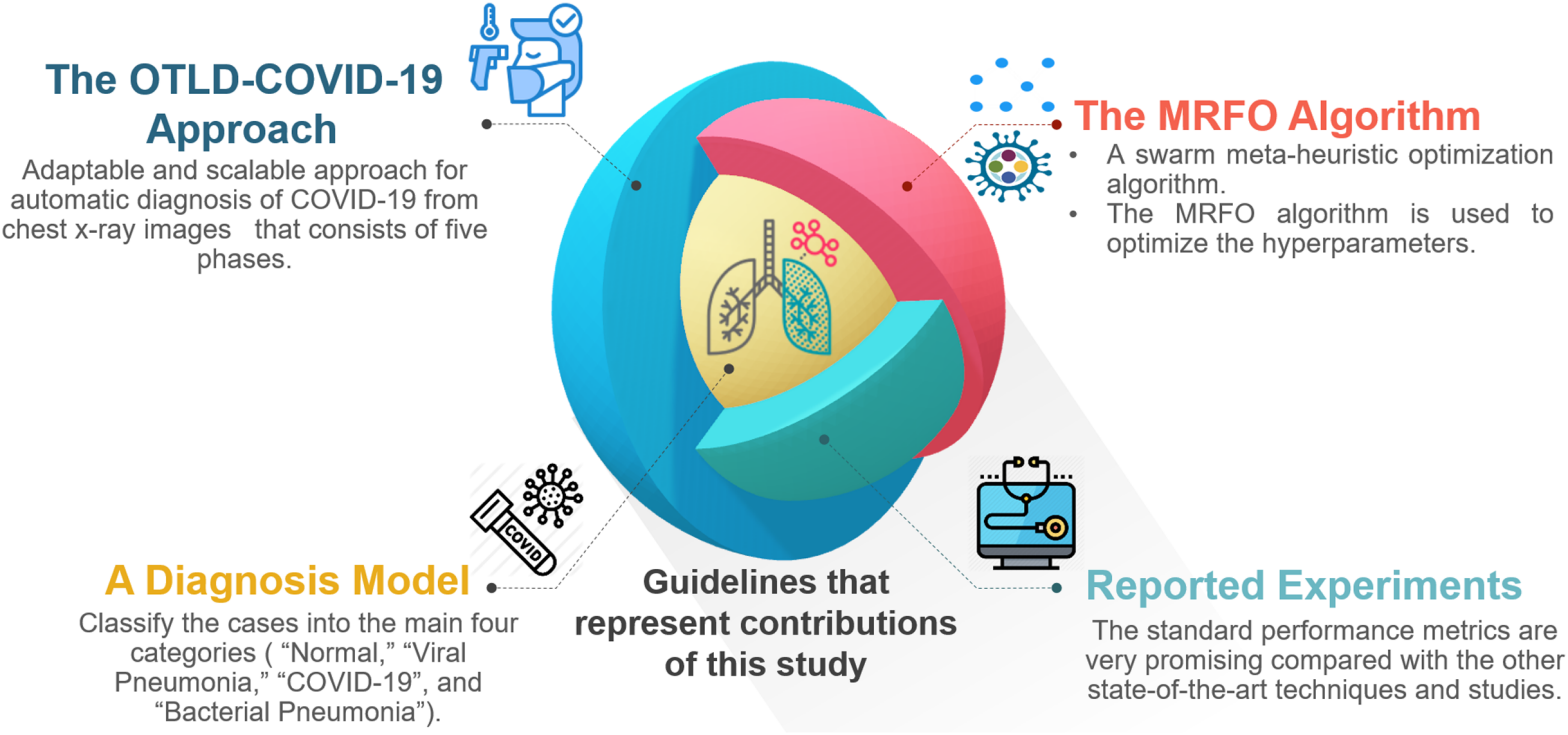
The guidelines that represent the contributions of this study.

For the convenience of readers, Fig. 3 depicts the guidelines of the four primary sections that reflect the contributions of this study. First, “The Proposed OTLD-COVID-19 Approach” introduces the proposed OTLD-COVID-19 approach. The OTLD-COVID-19 approach is characterized by its adaptability and scalability. It consists of five phases: (1) Acquisition phase, (2) Preprocessing phase, (3) Augmentation phase, (4) Training, Classification, and Optimization (TCO) phase, and (5) Deployment phase. Second, “The Proposed OTLD-COVID-19 Approach” illustrates the MRFO algorithm, which uses various foraging mathematical models to optimize the key hyperparameters’ values automatically. Third, the deployment phase of the proposed approach uses computed hyperparameters to build a diagnostic model. Each model evaluates the COVID-19 dataset to classify the cases into the main four categories (i.e., “COVID-19,” “Bacterial Pneumonia,” “Viral Pneumonia,” and “Normal”). Fourth, in “Experiments, Results and Discussion”, different reported experiments are introduced. The achieved results of the standard performance metrics are very promising compared with the other state-of-the-art techniques and studies.

In a summary, our OTLD approach improves the network performance in several ways. First, due to the lack of COVID 19 data, the proposed dataset is created from eight different datasets to increase the amount of data and avoid overfitting. Second, the MRFO optimization algorithm is used to obtain the optimal values of network hyperparameters. MRFO overcomes the main drawbacks of well-known metaheuristic algorithms, such as slow search speed and slight premature convergence. MRFO outperforms the other optimization algorithms in terms of search precision, convergence rate, stability, and local optimal value avoidance. Third, Transfer Learning (TL) is used to achieve the best network performance. Here, the MRFO optimization algorithm is applied to twelve different CNN architectures to obtain the best combination of hyperparameters for each architecture and obtain the architecture with the best performance. Finally, many performance metrics are measured to ensure the competence of the architecture with the best performance.

## Background

### CNN architectures

There is a continuous research investigation in the CNN architectures, and, notably, the noteworthy achievement in the CNN performance happened from 2015 to 2019. Khan et al. (2019) classified the CNN architectures into seven main categories. In this section, the most applicable CNN architectures from the mentioned classes are described. AlexNet (Antonellis et al., 2015) is designed to be the first known deep CNN architecture. AlexNet increases CNN’s depth by applying many different parameter optimization strategies to improve the learning capacity of the CNN (Antonellis et al., 2015). Although increasing the depth enhanced the generalization for different image resolutions, it caused the network to suffer from overfitting problems. Krizhevsky, Sutskever & Hinton (2012) adopted the idea of Hinton (Dahl, Sainath & Hinton, 2013; Srivastava et al., 2014) to solve the overfitting problem. They enforced the model to learn more robust features through skipping some transformational units randomly during the training process. Moreover, they used the Rectified Linear Unit (ReLU) activation function to enhance the convergence rate by mitigating the vanishing gradient issue (Nair & Hinton, 2010).

Simonyan & Zisserman (2014) proposed VGG, a CNN architecture designed simply and efficiently. It has 19 layers deeper than AlexNet to simulate the relationship between the network depth and capacity (Antonellis et al., 2015; Hodan, 1954). VGG addressed the large-size filter effect by replacing the large-size filter with a stack of (3 × 3) filters. Using the small-size filters enhanced the computation complexity. Unfortunately, the VGG still had a huge number of parameters, leading to severe difficulties in deploying it on low resource systems. Wu, Zhong & Liu (2018) proposed the CNN’s residual learning principle and efficiently trained the deep networks. ResNet presented a deeper network with less computational complexity than the previously proposed networks. ResNet is deeper than AlexNet and VGG by 20 and eight times, respectively (Simonyan & Zisserman, 2014).

The GoogleNet architecture was designed mainly to enhance the accuracy by reducing the computational cost (Szegedy et al., 2015). For this reason, the CNN inception block principle is presented where the conventional layers are replaced in small blocks (Lin, Chen & Yan, 2013). Each block has different size filters to get the spatial data at diverse scales. Inception-V3, Inception-V4, and Inception-ResNet are modified and enhanced versions of Inception-V1 and Inception-V2 (Roos & Schuttelaars, 2015; Szegedy et al., 2015; Szegedy et al., 2016). The main concept of Inception-V3 is to reduce the computational cost of deep networks without influencing the generalization. For that reason, Szegedy et al. (2016) used small and asymmetric filters ((1 × 5) and (1 × 7)) instead of large size filters ((5 × 5) and (7 × 7)). Besides, they used (1 × 1) convolution as a bottleneck in the front of the large filters. In Inception-ResNet, Szegedy et al. (2016) combined both the power of residual learning and inception block.

DenseNet was mainly designed to handle the vanishing gradient issue. ResNet specifically holds information through additive identity transformations; it suffers from a series problem, resulting in several layers that can contribute very little or no information (Blei, Ng & Jordan, 2003). DenseNet employed cross-layer connectivity differently. A feed-forward fashion is used to connect each previous layer to the next coming layer. The network’s information flow is substantially improved as DenseNet used the loss function to grant each layer direct access to the gradients. Xception is known to be an extreme Inception architecture, where the concept of a depth-wise separable convolution is embraced (Chollet, 2017) Xception achieved significant enhancements since it broadened the original inception block and regulated the computational complexity. The different spatial dimensions ((1 × 1), (5 × 5), and (3 × 3)) are replaced by a single dimension (3 × 3) followed by a (1 × 1) convolution. Also, Xception convolved each feature map in a separate way to get easier computations.

ResNet extended and improved the Inception Network (Xie et al., 2017). The authors applied the concept of splitting, transforming, and merging efficiently and simply. Besides, the cardinality concept is introduced (Szegedy et al., 2015). Cardinality is an extra dimension, which refers to the size of the set of transformations (Han et al., 2018; Sharma & Muttoo, 2018). The Inception network enhanced the convolution CNNs learning capability and ensured the efficient use of network resources. MobileNet (Howard et al., 2017) is a recent class of deep learning architectures explicitly designed for quick inference on mobile devices. MobileNets and other conventional models’ key difference is that two more efficient stages are added than the standard convolutional operation is decomposed into two more efficient stages. The depthwise separable convolutions are used to perform a single convolution on each color channel rather than combining all three and flattening it. In MobileNet V1, the pointwise convolution either kept the number of channels the same or doubled them. At the same time, MobileNetV2 (Sandler et al., 2018) decreases the number of channels. Table 1 compares the various CNN architectures.

**Table 1:** The CNN architectures comparison.

Architecture	Parameters	Error rate	Category
**AlexNet**	60 M	ImageNet: 16.4	Spatial Exploitation
**VGG**	138 M	ImageNet: 7.3	Spatial Exploitation
**GoogLeNet**	4 M	ImageNet: 6.7	Spatial Exploitation
		ImageNet: 3.5	
**Inception-V3**	23.6 M	Multi-Crop: 3.58	Depth + Width
****		Single-Crop: 5.6	
**Inception-V4**	35 M	ImageNet: 4.01	Depth +Width
**Inception-ResNet**	55.8 M	ImageNet: 3.52	Depth + Width + Multi-Path
**ResNet**	25.6 M	ImageNet: 3.6	Depth + Multi-Path
****	1.7 M	CIFAR-10: 6.43	
**Xception**	22.8 M	ImageNet: 0.055	Width
		CIFAR-10: 3.58	
		CIFAR-100: 17.31	
**ResNeXt**	68.1 M	ImageNet: 4.4	Width
	25.6 M	CIFAR-10+: 3.46	
**DenseNet**	25.6 M	CIFAR100+: 17.18	Multi-Path
	15.3 M	CIFAR-10: 5.19	
****	15.3 M	CIFAR-100: 19.64	
**MobileNet-V1**	4.2 M	ImageNet: 10.5	Depth + Width
**MobileNet-V2**	3.5 M	ImageNet	Depth + Width

### Transfer learning and CNN hyperparameters

Transfer learning is an effective representation learning approach in which the learned knowledge gained from a certain mission is used to enhance generalization about another. Transfer learning is much recommended when the number of images from the available datasets is relatively small. In this case, the original architecture and its weights are preserved and can be reused, especially when the used dataset in training the original architecture is vast. For example, the network architectures trained on the ImageNet dataset such as VGG (Simonyan & Zisserman, 2014) and DenseNet (Blei, Ng & Jordan, 2003) are extremely useful in medical image processing since it keeps the features of medical images in the ImageNet dataset. There are two common strategies to apply transfer learning: feature extraction and fine-tuning. In the feature extraction strategy, the last feed-forward layer(s) of the network is frozen. So, not all the weights are optimized; only the newly added layers are optimized during training. In the fine-tuning strategy, the pre-trained network is used as a starting point, and none of the weights are frozen, so all the network weights are optimized for the new task (Alshazly et al., 2019; Azizpour et al., 2015). When a fine-tuning strategy is adopted, it is recommended to apply lower learning rates to the pre-trained network to avoid the initial weights’ destruction.

Hyperparameters tuning is essential since it controls the overall behavior of a learning model. The main objective of tuning the hyperparameters is to get an optimal combination of hyperparameters that minimizes a predefined loss function to improve the network performance. CNN hyperparameters are classified into two main categories. The hyperparameters related to the network structure and The hyperparameters that determine how the network is trained. The main hyperparameters of both categories are described in Table 2.

**Table 2:** The CNN hyperparameters.

Category	Hyperparameters	Definition
Network Structure	Hidden layers	It represents the number of layers between the input and output layer.
	Kernel Size	It indicates the height and width of the 2D convolution window.
	Kernel Type	It specifies the applied filter (e.g. edge detection, sharpen).
	Stride	It specifies the step size of the kernel when crossing the image.
	Padding	The extra pixels of filler around the boundary of the input image that are set to zero.
	Dropout	It defines the percentage of neurons that should be ignored to prevent overfitting.
	Activation Functions	They are the mathematical equations that allow the model to learn nonlinear prediction boundaries.
Training Methodology	Learning Rate	It defines how quickly a network updates its parameters.
	Momentum	It specifies the value to let the previous update affect the current weight update.
	The Epochs Number	The number of iteration when the dataset is trained.
	Batch Size	It defines the number of patterns applied to the network before the weights are updated.
	Optimizer	It defines the parameters updating technique.

Several methods are used to determine the hyperparameters’ values, including manual search, grid search, random search, and optimization techniques. This paper proposes using the MRFO optimization technique to find out the values of the hyperparameters.

### Manta ray foraging optimization

Manta Ray Foraging Optimization (MRFO) (Zhao, Zhang & Wang, 2020) is a swarm meta-heuristic optimization algorithm bio-inspired by a foraging strategy practiced by the manta rays to capture the prey. Manta rays have developed various powerful and intelligent foraging strategies, such as chain foraging, cyclone foraging, and somersault foraging. Chain foraging mimics the intrinsic behavior of the food search. Foraging manta rays line up in an organized fashion to capture lost preys missed or unnoticed by the chain’s previous manta rays. This cooperative interaction between rival manta rays decreases the risk of prey loss while also increasing food rewards. Cyclone foraging occurs when there is a high density of prey. The tail end of the manta ray connects with its head forming a spiral to produce a vertex in the eye of a cyclone, causing the filtered water to rise to the surface. This complex mechanism allows a manta ray to capture the prey easily. The last foraging strategy is the somersault foraging. When manta rays find a food source (plankton), they perform backward somersaults before circling around the plankton, pulling it towards them. These foraging behaviors are extremely successful, despite their rarity in nature. The following sections cover the mathematical models for each foraging strategy. It worth mentioning that the used symbols in the current study for the MRFO models are similar to the MRFO original paper to avoid misunderstandings.

#### Chain foraging

**Figure 4:**
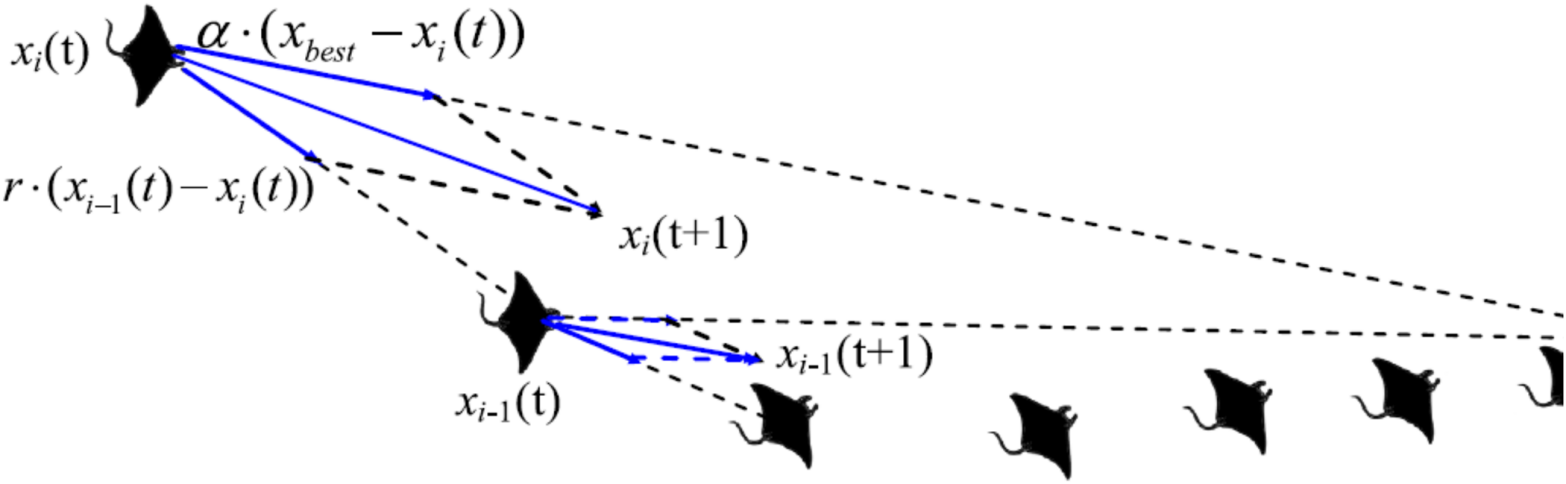
Chain foraging behavior in the 2-D space illustration (Zhao, Zhang & Wang, 2020).

Manta rays hunt for prey plankton and swim towards it after determining its location. The best location is one with a higher plankton concentration. Manta rays form a foraging chain by forming a line from head to tail. Each manta ray adjusts its position based on the best solution achieved so far and the location of the one in the front. Figure 4 shows the chain behavior.

#### Cyclone foraging

**Figure 5:**
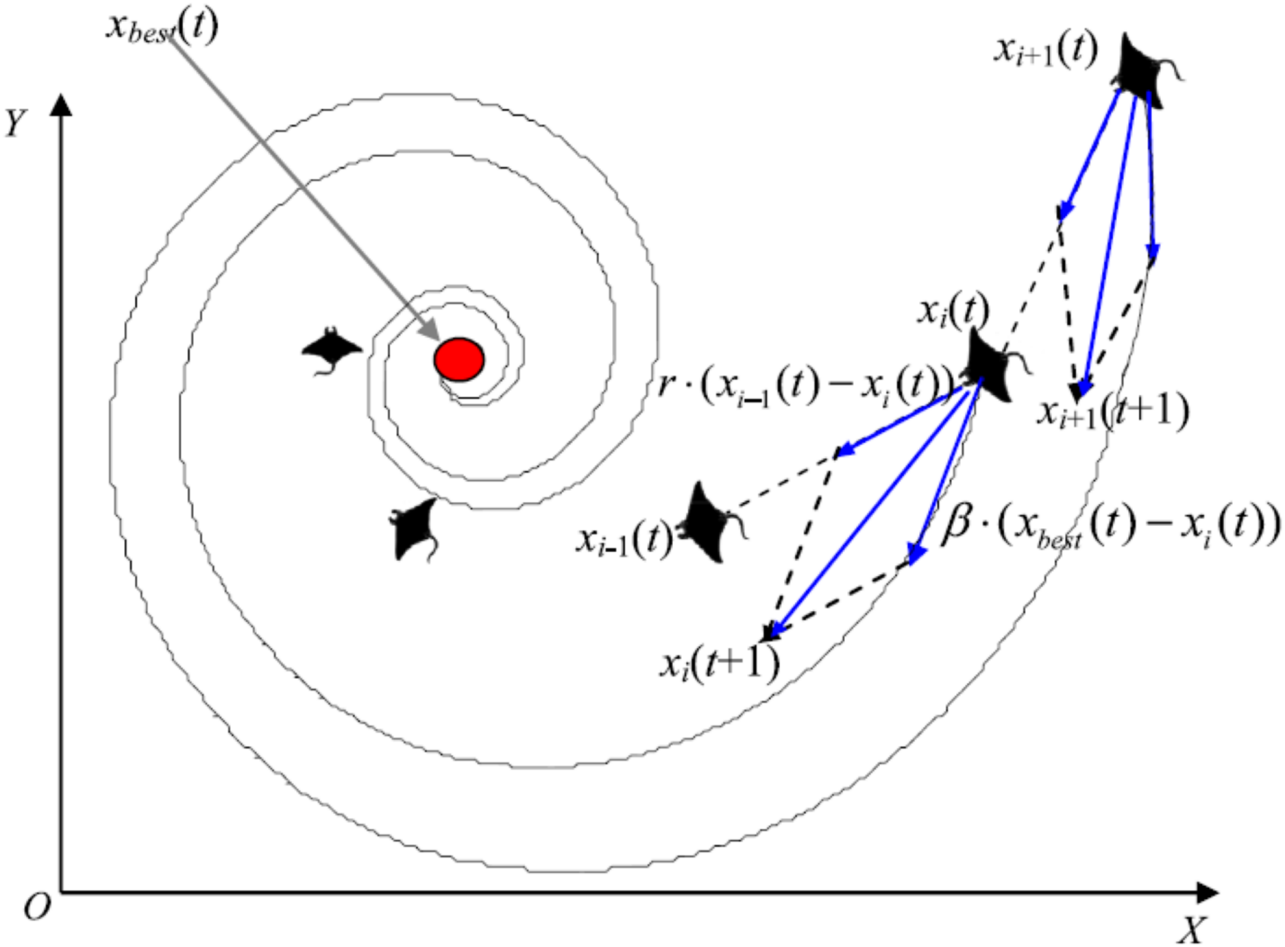
Cyclone foraging behavior in the 2-D space illustration (Zhao, Zhang & Wang, 2020).

When a group of manta rays recognizes a plankton group in deep water, they form a foraging chain and make spiral movements as they approach the food source. During the cyclone foraging process, flocked manta rays not only pursue the manta ray in front of them to ensure the chain’s continuity, but they also chase a spiral pathway to get to the target prey. Figure 5 shows the cyclone behavior.

#### Somersault foraging

**Figure 6:**
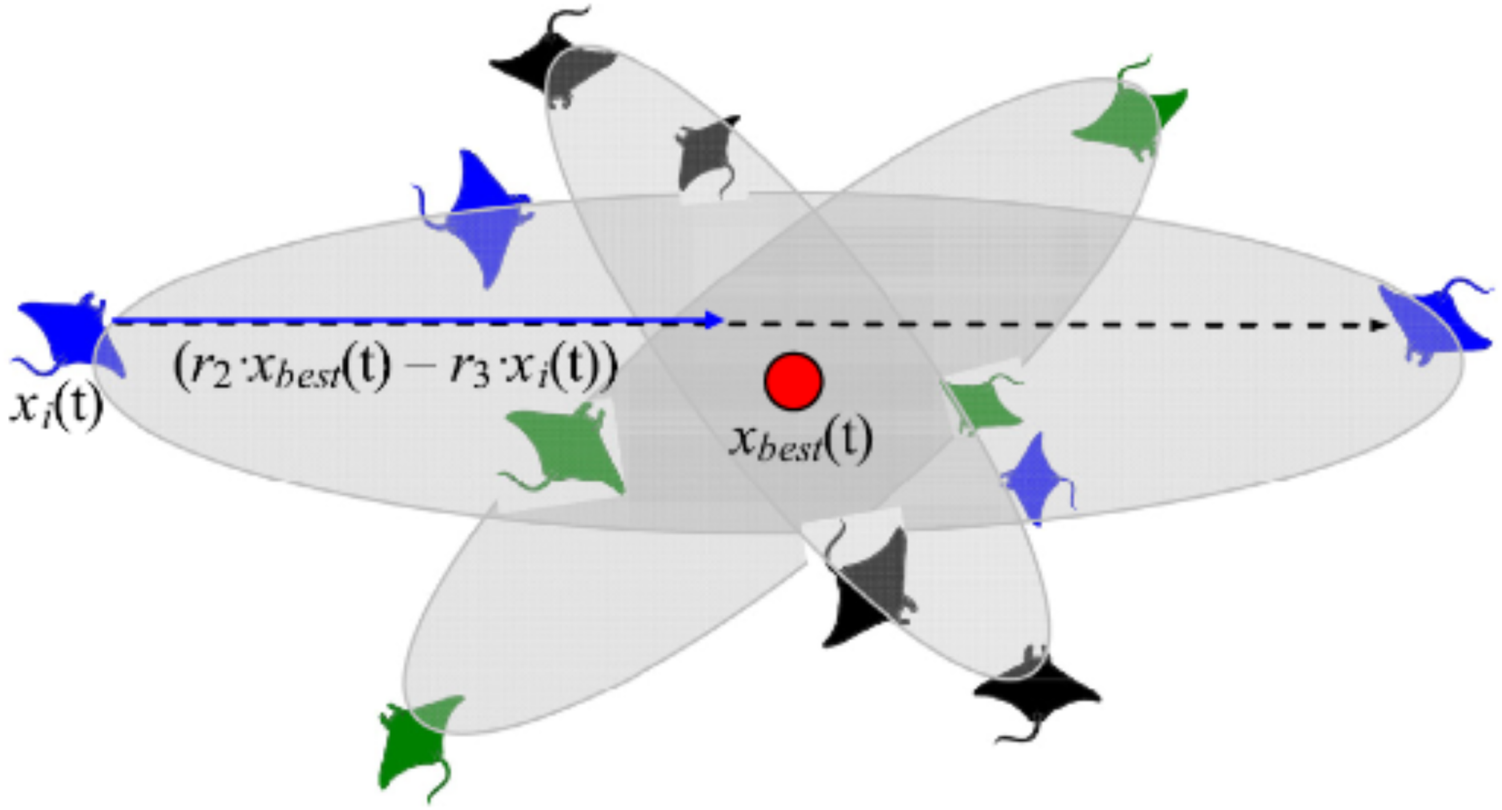
Somersault foraging behavior in the MRFO illustration (Zhao, Zhang & Wang, 2020).

This foraging scheme considers the best prey location as a pivot point. Each manta-ray in the population searches around this point to migrate to a new location in the search domain. In the search space, all individuals progressively approximate to the optimal solution. As a result, the range of somersault foraging is reduced as iterations increase. Figure 6 demonstrates the pattern of somersault foraging in MRFO.

## Related work

Convolutional Neural Network (CNN) is considered one of the most effective deep learning approaches for accurately analyzing medical images. The main features to identify the COVID-19 in medical images include bilateral distribution of patchy shadows and ground-glass opacity (Wang et al., 2020). The research effort in the COVID-19 detection can be classified into three different perspectives related to deep learning techniques. These perspectives are tailored to CNN, transfer learning, and hybrid architectures. This section discusses the research studies to automate the detection of the COVID-19 according to these perspectives.

A tailored CNN Architectures is a CNN network designed specifically to detect the COVID-19. The network is trained for the time. Mukherjee et al. (2020) introduced introduced a shallow CNN architecture consisting of four layers. Light architecture is to minimize the number of parameters (i.e., weights) to speed up computational time. Besides, the shallow (or lightweight) CNN design avoids possible overfitting that faces architectures with heavy usage of parameters. The shallow CNN architecture is well fit for mass population screening, especially in resource-constrained areas. The tailored shallow CNN model is designed to diagnose 2 class classification (“COVID” and “Normal”). It achieved an accuracy, sensitivity, and (Area Under Curve) AUC of 96.92%, 0.942, and 0.9869 respectively.

Wang, Lin & Wong (2020) introduced COVID-Net, a tailored deep CNN architecture for detecting the COVID-19 cases using CXR images. They claimed that COVID-Net is one of the first open-source network designs for COVID-19 detection. They also introduced COVIDx, an open-access benchmark dataset with 13,975 CXR images representing 13,870 patient cases. The COVIDx dataset is generated by combining and modifying five open access existing datasets having chest scans. Their experimental results showed the achieved accuracy is 92.4% to classify “Normal,” “non-COVID Pneumonia,” and “COVID-19” classes.

Majeed et al. (2020) introduced CNN-X, a tailored CNN architecture that holds four parallel layers. Each layer has 16 filters in three different sizes (3 × 3), (5 × 5), and (9 × 9). (3 × 3) filters detect the local-features while global features are detected by (9 × 9) filter. The (5 × 5) filter is used to detect what is missed by the other two filters. Then, the convolved image is applied to batch normalization and a ReLU activation function. Afterward, average pooling and maximum pooling are applied. The reason for using different size filters is to detect. They used Two COVID-19 X-ray image datasets in addition to a large dataset for other infections.

Hammoudi et al. (2020) proposed a tailored CNN architecture to handle the two data modalities (CT and X-rays). Their model consists of nine layers for detecting COVID-19 positive cases. They trained and tested their network using both CT scans and X-rays. The experimental results show that their architecture achieved an overall accuracy of 96.28%.

Hussain et al. (2021) presented a CoroDet, a tailored 22-layer CNN architecture to detect COVID-19 using both chest X-ray and CT modalities. The architecture consists of several layers: convolution layer, pooling layer, dense layer, flatten layer, and three activation functions. The CoroDet is designed to diagnose 2 class classification (“COVID-19” and “Normal”), 3 class classification (“COVID-19”, “Normal”, and “Pneumonia”), and 4 class classification (“COVID-19”, “Normal”, “non-COVID Viral Pneumonia”, and “non-COVID Bacterial Pneumonia”). Their architecture’s accuracy varies from 99.1% for the two classes to 91.2% for the four classes classification.

Traditional transfer learning strategies are promising since the COVID-19 pneumonia CXR data is very limited. In this work, the popular deep learning architectures are customized to the purpose of the COVID-19 detection. Only the last few layers of the pre-trained model are replaced and retrained. The modified CNN architecture gets the advantages from the base CNN. The learned feature representations are fine-tuned to improve performance. Farooq & Hafeez (2020) suggested using the ResNet50 architected. They replaced the head of the trained model with another head containing a sequence of Adaptive average/max pooling, batch normalization, drop out, and linear. The ResNet50 weights are pre-trained using the ImageNet dataset that has X-ray images with different sizes.

Apostolopoulos & Mpesiana (2020) tested five standard CNN architectures, including VGG19, InceptionNet, MobileNetV2, XceptionNet, and Inception. Different hyperparameters are used to identify the COVID-19. They used two different datasets having X-ray images from public medical repositories. Their results showed that the best-achieved accuracy, sensitivity, and specificity are 96.78%, 98.66%, and 96.46%, respectively, obtained from MobileNetV2 architecture. Narin, Kaya & Pamuk (2020) used five pre-trained convolutional neural network-based models to identify the COVID-19 using chest X-ray images. They implemented three different binary classifications with four classes (“COVID-19”, “Normal” (i.e., “Healthy”), “Viral Pneumonia,” and “Bacterial Pneumonia”). The results showed that the pre-trained ResNet50 model achieves the best classification performance.

Islam et al. (2020) performed modality-specific transfer learning through retraining the ImageNet Network on the RSNA CXR collection to learn CXR modality-specific features and detect the abnormality. The used collection contains both normal CXRs and abnormal images having pneumonia-related opacities. Dropout is used to overcome overfitting where the regularization is restricted, and generalization is improved by reducing the model sensitivity to the training input’s specifics. The different hyperparameters of the CNNs are optimized using a randomized grid search method.

Nayak et al. (2021) introduced a deep Learning architecture to automate the COVID-19 detection using X-ray images. They evaluated the performance of eight CBB architectures to detect COVID-19 cases. The introduced architectures are compared by considering several different hyperparameters values. The results showed that the ResNet-34 model achieved a higher accuracy of 98.33%. Khan, Shah & Bhat (2020) presented CoroNet architecture, a pre-trained CNN architecture to detect COVID-19 pneumonia from three different kinds of pneumonia using CXR images. The CNN architecture relies on Exception (Extreme Inception) and contains 71 layers trained on the ImageNet dataset. The authors introduced a balanced dataset containing 310 normal, 330 bacterial, 327 virals, and 284 COVID-19 resized CXR images. The experimental results showed that the CoroNet architecture achieved an accuracy of 0.87 and an F1-score of 0.93 for the COVID-19 detection.

In most typical deep learning architectures, the CNN is used for both feature extraction and classification. Combined architectures use CNN either for feature extraction and apply another classifier to identify the COVID-19 patients or classify and use other algorithms to extract and optimize features. The hybrid architecture combines different deep learning algorithms or combines deep learning with other AI models such as machine learning and data mining.

Islam, Islam & Asraf (2020) introduced a deep learning architecture that combines a CNN and a Long Short-term Memory (LSTM) to identify the COVID-19 from X-ray images automatically. They used the CNN network to extract deep features and LSTM for the detection of the COVID-19 patients. Another study (Islam et al., 2020) introduced architecture to diagnose the COVID-19 using chest X-rays. The architecture combined CNN for feature extraction and recurrent neural network (RNN) for classification to diagnose the COVID-19 from chest X-rays. They used many deep transfer techniques, including VGG19, DenseNet121, InceptionV3, and InceptionResNetV2. They showed that the performance of VGG19-RNN is better than the other compared architectures in terms of accuracy and computational time in our experiments.

Aslan et al. (2021) presented a hybrid architecture that combines the CNN and Bi-directional Long Short-term Memories (BiLSTM). They utilized the modified AlexNet (mAlexNet) architecture with chest X-ray images to diagnose the COVID-19. They modified the last three layers of the AlexNet model to build a three-class model classify the COVID-19. The remaining parameters of the original AlexNet model have been preserved. The temporal features obtained from the BiLSTM layer are passed as input to a fully-connected (FC) layer, and the Softmax is used for the classification.

Sethy et al. (2020) proposed a hybrid architecture that relies on ImageNet pre-trained models to extract the high-level features and Support Vector Machine (SVM) to detect the COVID-19 cases. Their architecture is a three-class problem to classify the COVID-19 patient from healthy people and pneumonia patients using X-ray images. They showed that the SVM achieved the best results when the features are extracted using the ResNet50 Network.

Altan & Karasu (2020) presented a combined architecture to detect the COVID-19 patients from X-ray images. The architecture consists of three different techniques: two-dimensional (2D) curvelet transformation, Chaotic Salp Swarm Algorithm (CSSA) optimization algorithm, and deep learning technique. 2D Curvelet transform is used to get the feature matrix from the patient’s chest X-ray images. An optimization process is done to the feature matrix. The EfficientNetB0 model, based on CNN, is used to classify X-ray images to diagnose the infected COVID-19.

## The proposed otld-covid-19 approach

Recently, the COVID-19 pandemic has taken the world’s health care systems by surprise. It will also take a long time to ensure the vaccine’s safety before the general public could use it. As a result, the government’s existing efforts primarily focus on preventing the spread of COVID-19 and forecasting potential pandemic areas. Due to the scarcity of COVID-19 testing kits, particularly in developing countries, alternative diagnosis methods are essential. One solution is to develop COVID-19 diagnosis strategies based on data mining and artificial intelligence.

**Figure 7:**
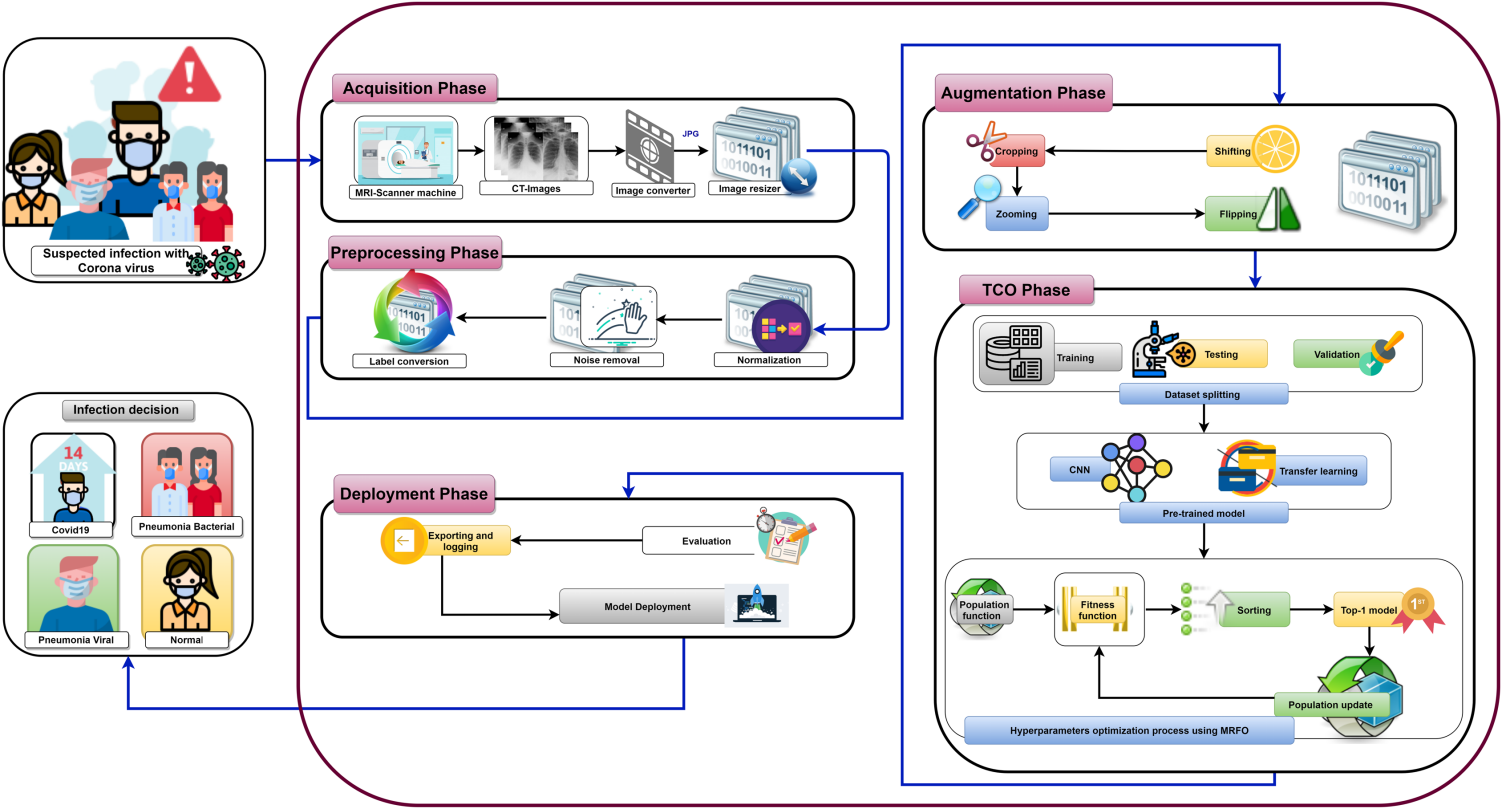
The proposed OTLD-COVID-19 approach.

Figure 7 shows the proposed OTLD-COVID-19 approach that consists of five phases: (1) Acquisition phase, (2) Preprocessing phase, (3) Augmentation phase, (4) Training, Classification, and Optimization (TCO) phase, and (5) Deployment phase. The acquisition phase starts with reading the dataset, converting images to the “JPG” format, and resize the images to (64, 64, 3). Normalizing the images *X* (i.e., $\frac{X}{255.0}$) is performed in the preprocessing phase, followed by noise removal and converting the labels (i.e., classes) from numeric to one-hot encoding. In one-hot encoding, each category is converted into a new column and assigned a 1 or 0 as notation for true/false (e.g. 2 will be [0, 1, 0, 0] for 4 classes).

The first stage of data augmentation aims to equalize the number of images in each category via shifting, cropping, zooming, and flipping (horizontally, vertically, or both). The TCO phase is the main core of the proposed approach. The first stage splits the augmented dataset into three parts (training, testing, and validation). It applies the transfer learning to obtain a pre-trained CNN model with ImageNet. In this study, twelve different models are tested to create the pre-trained CNN model (i.e., DenseNet121, DenseNet169, DenseNet201, Xception, MobileNet, MobileNetV2, MobileNetV3Small, MobileNetV3Large, EfficientNetB0, ResNet50V2, ResNet101V2, and ResNet152V2). The second data augmentation stage is applied during the training. The training optimizes the learning parameters. The last stage in this phase is the hyperparameters optimization process.

The MRFO algorithm is repeated for several iterations (i.e., 15 in the current study) to optimize the main hyperparameters. It defines the population size (*N*_*p*_), maximum number of iterations (*N*_*s*_) and dataset split ratio (*SR*). Random positions of the manta-rays are initialized before applying either chain or cyclone foraging according to a random value. MRFO starts the chain foraging if the random value is lower than 0.5. The chain foraging mathematical model is represented in Eqs. (1) and (2) as follows:

(1) $$x_{i}^{d}(t+1)=(\begin{array}{cc} x_{i}^{d}(t)+r\times (x_{best}^{d}(t)-x_{i}^{d}(t))+\alpha \times (x_{best}^{d}(t)-x_{i}^{d}(t)) & i=1\\ x_{i}^{d}(t)+r\times (x_{i-1}^{d}(t)-x_{i}^{d}(t))+\alpha \times (x_{best}^{d}(t)-x_{i}^{d}(t)) & i=2,\ldots ,N \end{array}$$

(2) $$\alpha =2\times r\times \sqrt{\left|\log(r)\right|}$$

where $x_{i}^{d}(t)$ is the position of *i*^*th*^ individual at time *t* in *d*^*th*^ dimension, *r* is a random vector within the range of [0, 1], *α* is a weight coefficient, and $x_{best}^{d}(t)$ is the plankton with the highest concentration.

The cyclone foraging process plays an important part in developing two key driving mechanisms: exploitation and exploration. The exploitation (intensification) aims to find the best candidate solutions in the current search space, called the local search. The exploration (the global search) is concerned with exploring different search space areas to avoid getting stuck in a local minimum. In this foraging process, the best plankton location is used as a reference point, allowing for increased exploitation capabilities by enlarging the fertile regions surrounding the current best solution. Eqs. (3) and (4) mathematically models the exploitation phase.

(3) $$x_{i}^{d}(t+1)=(\begin{array}{cc} x_{best}^{d}+r\times (x_{best}^{d}(t)-x_{i}^{d}(t))+\beta \times (x_{best}^{d}(t)-x_{i}^{d}(t)) & i=1\\ x_{best}^{d}+r\times (x_{i-1}^{d}(t)-x_{i}^{d}(t))+\beta \times (x_{best}^{d}(t)-x_{i}^{d}(t)) & i=2,\ldots ,N \end{array}$$

(4) $$\beta =2\times \exp^{r_{1}\times \left(\frac{T-t+1}{T}\right)}\times \sin(2\times \pi \times r_{1})$$

where *β* is the weight coefficient, *T* is the maximum number of iterations, and *r*_1_ is a random number in the range [0, 1].

Cyclone foraging helps the exploration process by forcing the population members to shift to a random location in the search space. This position is far from their current location as well as the best prey location. This exploration method helps the algorithm extend the global search space and direct the population through the search domain’s unvisited paths. The mathematical model of the exploration process is given by Eqs. (5) and (6) below.

(5) $$x_{rand}^{d}=LB^{d}+r\times (UB^{d}-LB^{d})$$

(6) $$x_{i}^{d}(t+1)=(\begin{array}{cc} x_{rand}^{d}+r\times (x_{rand}^{d}(t)-x_{i}^{d}(t))+\beta \times (x_{rand}^{d}(t)-x_{i}^{d}(t)) & i=1\\ x_{rand}^{d}+r\times (x_{i-1}^{d}(t)-x_{i}^{d}(t))+\beta \times (x_{rand}^{d}(t)-x_{i}^{d}(t)) & i=2,\ldots ,N \end{array}$$

where $x_{rand}^{d}$ is a random position randomly produced in the search space, and *LB*^*d*^ and *UB*^*d*^ are the lower and upper limits of the *d*^*th*^ dimension, respectively.

MRFO shifts between exploration phases according to the ratio between current iteration and the maximum number of iterations (*n*_*s*_/*N*_*s*_). The exploitation phase is enacted when *n*_*s*_/*N*_*s*_<*rand*. The technique switches to the exploration phase if *n*_*s*_/*N*_*s*_>*rand*.

After completing either cyclone or chain foraging, summersault foraging takes action. It updates the current position of individuals through the current best solution. The following mathematical formulation (i.e., Eq. (7)) describes the summersault foraging.

(7) $$x_{i}^{d}(t+1)=x_{i}^{d}(t)+S\times (r_{2}\times x_{best}^{d}-r_{3}\times x_{i}^{d}(t)),i=1,\cdots ,N$$

where *S* is the somersault factor that decides the somersault range of manta rays and its value equals 2, *r*_2_ and *r*_3_ are two random numbers in the range [0, 1].

The TCO phase computes the fitness score of each manta-ray and chooses the best individual. The metrics are determined from the evaluation of the trained model on the test part of the dataset. These metrics are used to calculate the overall fitness score after applying the trained model for several epochs (i.e., 64 in the current study). The calculated fitness score is used to update the position of manta-rays. This process repeats until the completion of the iterations (i.e. *n*_*s*_ = *N*_*s*_). Each result is evaluated, and a model is built according to the optimized computed hyperparameters. The model with these optimized hyperparameters is ready to achieve a rigid classification process. The pseudocode of the TCO is represented in Algorithm 1 where the “UpdateMRFO” function that uses the MRFO optimization algorithm is represented in Algorithm 2.

**Algorithm 1:** The TCO Pesudocode.

**Input:** *N*_*p*_, *N*_*s*_, *SR*, *Model* // Population Size, Number of Iterations, Dataset Split Ratio, CNN Pre-trained Model **Output:** *bestSoFar //* The Best Combination **Data:** *X*, *Y* // Images Dataset, Images Labels *POs* ⇐ The Learning Parameters Optimizers *BSs* ⇐ The Learning Batch Sizes *MLRs* ⇐ The Learning Ratios *DRs* ⇐ The Learning Dropout Ratios *Metrics* ⇐ The Learning Performance Metrics *n*_*s*_ ⇐ 1 // Initialize the Iterations Counter *fitnessScores* ⇐ [ ] *trainX*, *validationX*, *testX*, *trainY*, *validationY*, *testY* ⇐ *DatasetSplit*(*X*, *Y*, *SR*) // Get *N*_*p*_ Solutions using a Random Initialization Process *population* ⇐ *InitializePopulation*(*POs*, *BSs*, *MLRs*, *DRs*, *N*_*p*_) // Find the Population Best Combinations using the Iterative Optimization Approach **while** *n*_*s*_ ≤ *N*_*s*_ **do** // Find the Population Fitness Score Values of the Current Population *n*_*p*_ ⇐ 1 // Initialize the Populations Counter **while** *n*_*p*_ ≤ *N*_*p*_ **do** *solution* ⇐ *GetSolution*(*population*, *n*_*p*_) // Get the Solution at Index of *n*_*p*_ *Model* ⇐ *LearnCNN*(*Model*, *solution*, *Metrics*, *trainX*, *trainY*, *validationX*, *validationY*) // Apply the CNN Model Learning Proce (i.e. Train and Validate) *metrics* ⇐ *TestCNN*(*Model*, *Metrics*, *testX*, *testY*) // The the Optimized CNN using the Required Metrics *fitnessScore* ⇐ *GetOverallFitnessScore*(*metrics*) // Get the Overall Fitness Score using the Metrics’ Results *fitnessScores.push*(*fitnessScore*) // Append the Latest Fitness Score to the End of the List *n*_*p*_ ⇐ *n*_*p*_ + 1 // Increment the Populations Counter **end** *sortedPopulation* ⇐ *SortFitnessScores*(*population*, *fitnessScores*) // Sort the Population concrening the Fitness Score Values *population* ⇐ *UpdateMRFO*(*sortedPopulation*, *POs*, *BSs*, *MLRs*, *DRs*, *N*_*p*_) // Update the Population using the MRFO Algorithm *n*_*s*_ ⇐ *n*_*s*_ + 1 // Increment the Iterations Counter **end** *bestSoFar* ⇐ *GetTop*(*population*) // Extract the Overall Top Combination

**Algorithm 2:** The Manta Ray Foragin Optimization (MRFO) Algorithm Pesudocode.

**Input:** *population*, *bestFitness*, *N*_*s*_, *n*_*s*_, *LB*, *UB*, *D* // Population, The Best Fitness Score, Number of Iterations, Current Iteration, Lower Bound, Upper Bound, Number of Dimensions **Output:** *newPopulation* // The New Population *newPopulation* ⇐ *Copy*(*population*) Create a Copy from the Current Sorted Population *bestSolution* ⇐ *newPopulation* [1] Get the Best Solution (i.e., First Solution) From the Sorted Population *coef* ⇐ $\frac{n_{s}}{N_{S}}$ *n*_*p*_ ⇐ 1 Initialize the Populations Counter **while** (*n*_*p*_ ≤ *N*_*p*_) **do** *r* ⇐ *random*() // A Random Number in the Range [0, 1] *r*1 ⇐ *random*() // A Random Number in the Range [0, 1] α ⇐ 2.0 × *r* × $\sqrt{\left||\text{log}r|\right|}$ // Calculate the Value of Alpha *factor* ⇐ $\frac{N_{s}-n_{s}+1.0}{N_{S}\times 1.0}$ *β* ⇐ 2.0 × exp(*r*1 × *factor*) × sin(2.0 × *π* × *r*1) // Calculate the Value of Beta **if** (*random*() < 0.5) then // Cyclone Foraging **if** (*coef* < *random*()) **then** *rand* ⇐ *LB* + *random*(*D*) × (*UB* − *LB*) **if** (*n*_*p*_ = 1) **then** *newPopulation*[*n*_*p*_] ⇐ *rand* + *r* × (*rand* − *newPopulation*[*n*_*p*_]) + *β* × (*rand* − *newPopulation*[*n*_*p*_]) **end** **else** *newPopulation*[*n*_*p*_] ⇐ *rand* + *r* × (*newPopulation*[*n*_*p*_ − 1] − *newPopulation*[*n*_*p*_]) + *β* × (*rand* − *newPopulation*[*n*_*p*_]) **end** **end** **else** **if** (*n*_*p*_ = 1) **then** *newPopulation*[*n*_*p*_] ⇐ *bestSolution* + *r* × (*bestSolution* − *newPopulation*[*n*_*p*_]) + *β* × (*bestSolution* − *newPopulation*[*n*_*p*_]) **end** **else** *newPopulation*[*n*_*p*_] ⇐ *bestSolution* + *r* × (*newPopulation*[*n*_*p*_ − 1] − *newPopulation*[*n*_*p*_]) + *β* × (*bestSolution* − *newPopulation*[*n*_*p*_]) **end** **end** **end** **else** // Chain Foraging **if** *n*_*p*_ = 1 **then** *newPopulation*[*n*_*p*_] ⇐ *newPopulation*[*n*_*p*_] + *r* × (*bestSolution* − *newPopulation*[*n*_*p*_]) + *α* × (*bestSolution* − *newPopulation*[*n*_*p*_]) **end** **else** *newPopulation*[*n*_*p*_] ⇐ *newPopulation*[*n*_*p*_] + *r* × (*newPopulation*[*n*_*p*_ − 1] − *newPopulation*[*n*_*p*_]) + *α* × (*bestSolution* − *newPopulation*[*n*_*p*_]) **end** **end** *fitnessScore* ⇐ *GetFitnessScore*(*newPopulation*[*n*_*p*_]) // Get the Overall Fitness Score After Re-Learning the Model **if** (*fitnessScore* > *bestFitness*) **then** *bestSolution* ⇐ *newPopulation*[*n*_*p*_] *bestFitness* ⇐ *fitnessScore* **end** Somersault Foraging *S* ⇐ 2.0 *r*2 ⇐ *random*() *r*3 ⇐ *random*() *newPopulation*[*n*_*p*_] ⇐ *newPopulation*[*n*_*p*_] + *S* × (*r*2 × *bestSolution* − *r*3 × *newPopulation*[*n*_*p*_]) *fitnessScore* ⇐ *GetFitnessScore*(*newPopulation*[*n*_*p*_]) // Get the Overall Fitness Score After Re-Learning the Model **if** (*fitnessScore* > *bestFitness*) **then** *bestSolution* ⇐ *newPopulation*[*n*_*p*_] *bestFitness* ⇐ *fitnessScore* **end** **end**

The deployment phase of the proposed approach uses computed hyperparameters to build a classification model. Each model evaluates the COVID-19 dataset to classify the cases into the main four categories (i.e., “Normal,” “Viral Pneumonia,” “COVID-19”, and “Bacterial Pneumonia”). The next section will discuss the experimental results of the proposed OTLD-COVID-19 approach compared to recent state-of-the-art approaches and explain its effectiveness.

## Experiments, results and discussion

This section presents the different applied experiments with the corresponding results and discussions. It also presents the used dataset in the current study and ends by applying a comparative study between the current study and a set of state-of-the-art studies.

### Dataset and experiments configurations

**Figure 8:**
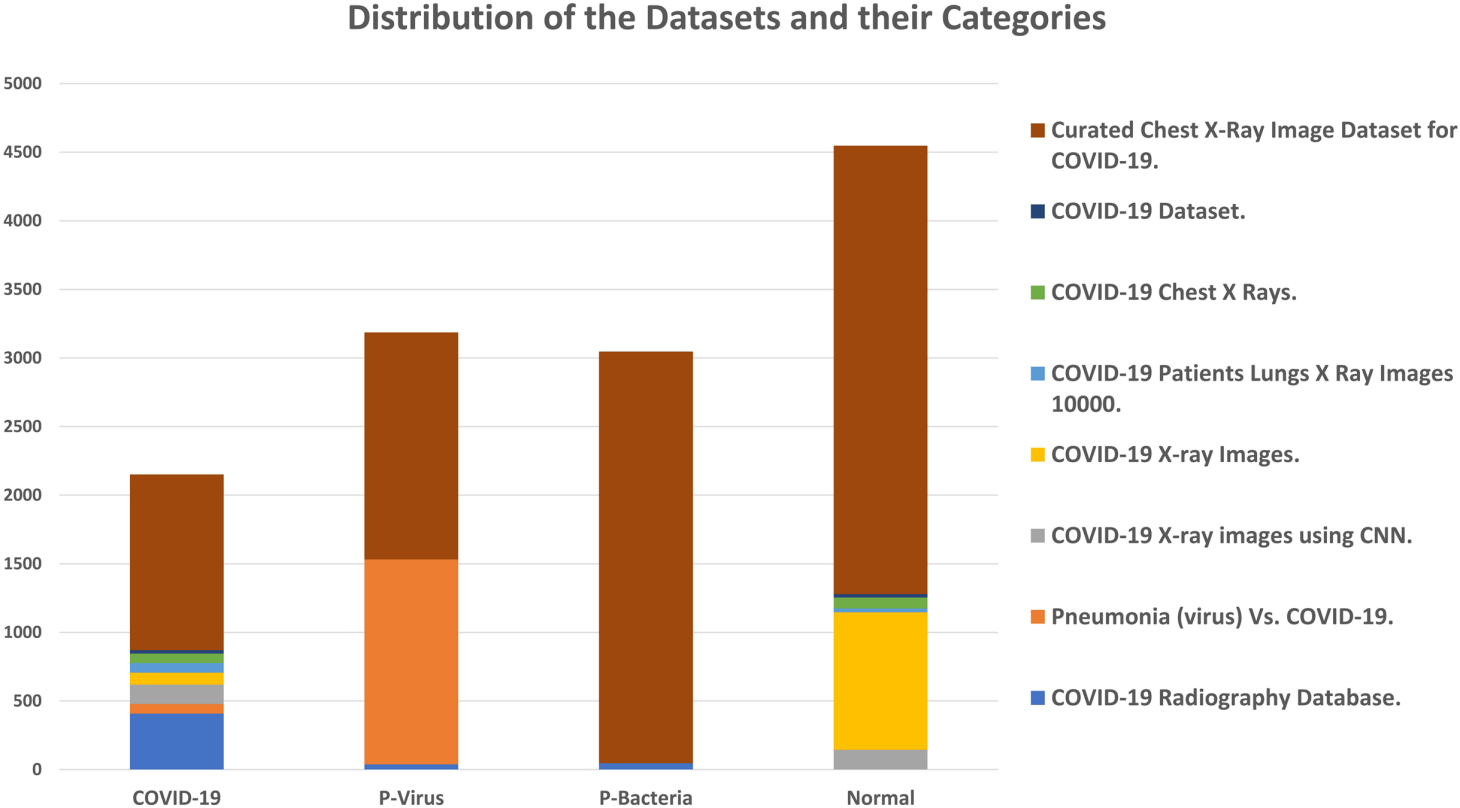
Distribution of the datasets and their categories.

The adopted datasets in this study are real datasets used to distinguish COVID-19 from common pneumonia types. The proposed dataset is unified and collected from eight different public data sources described in Table 3 and graphically illustrated in Fig. 8. The dataset consists of chest X-ray images in four classes: radiographs of normal cases, viral pneumonia, COVID-19 pneumonia, and bacterial pneumonia. The total number of cases in the collected dataset is 12,933. Table 4 summarizes the common experiments configurations.

**Table 3:** The used datasets description.

#	Dataset	COVID-19	P-virus	P-bacteria	Normal
1	COVID-19 Radiography Database (Cohen et al., 2020).	408	38	46	0
2	Pneumonia (virus) Vs. COVID-19 (Mahasin, 2020).	70	1,493	0	0
3	COVID-19 X-ray images using CNN (Srikar, 2020).	140	0	0	144
4	COVID-19 X-ray Images (Das, 2020).	88	0	0	1,002
5	COVID-19 Patients Lungs X Ray Images 10000 (Sajid, 2020).	70	0	0	28
6	COVID-19 Chest X Rays (Sreeraman, 2020).	69	0	0	79
7	COVID-19 Dataset (Riaz, 2020).	25	0	0	25
8	Curated Chest X-Ray Image Dataset for COVID-19 (Sait, 2020).	1,281	1,656	3,001	3,270
**Total**		2,151	3,187	3,047	4,548

**Table 4:** Experiments configurations summarization.

Key	Value
Dataset	8 Resources
Categories	“Normal”, “P-Viral”, “P-Bacterial”, and “COVID-19”
Dataset Size	12,933
Pre-trained Models	DenseNet121, DenseNet169, DenseNet201, Xception, MobileNet, MobileNetV2, MobileNetV3Small, MobileNetV3Large, EfficientNetB0, ResNet50V2, ResNet101V2, and ResNet152V2
Parameters Initializer	ImageNet
Parameters Optimizers	Adam, NAdam, Ftrl, AdaDelta, AdaGrad, AdaMax, RMSProp, and SGD
Output Activation Function	SoftMax
Model Learn Ratios	[0 : 5 : 100]%
Batch Sizes	[8 : 8 : 100]
Dropout Ratios	[1 : 1 : 60]%
Number of Epochs	8 Epochs
Performance Metrics	Accuracy, Loss, Precision, F1-score, AUC, Dice Coef., IoU Coef., Specificity, and Recall
Number of Iterations *N*_*s*_	20 Iterations
Population Size *N*_*p*_	10 Candidates
Split Ratio *SR*	85% to 15%
Image Size	(64, 64, 3)
Data Augmentation	Applied (in Two Stages)
Training Environment	Google Colab (using its GPU)

### Performance metrics

During the next experiments, there are different metrics to evaluate the performance of the “OTLD-COVID-19” approach. At first, the confusion matrix that represents a summary of predicted results is constructed. The confusion matrix has four values as follows:

- True Positive (TP) occurs when the actual class of the data is positive (True) and the predicted is also positive (True).
- True Negative (TN) occurs when the actual class of the data is negative (False), and the predicted is also negative (False).
- False Positive (FP) occurs when the actual class of the data is negative (False) while the predicted is positive (Tues).
- False Negative (FN) occurs when the actual class of the data is positive (True), and the predicted is negative (False).

Different formulas are used as a summarization of the confusion matrix. Table 5 depicts several performance metrics, including Accuracy, Recall, Precision, F1-score, and Loss. Among these metrics, accuracy has the most attention for the results of deep learning classifiers in the condition that the data is well balanced and not skewed for a specific class. It is the fraction of predictions the model classified correctly to all the predictions of the model. Precision is used as an evaluation metric to ensure our prediction. Recall or Sensitivity (True Positive Rate) is essential to understand how complete the results are. F1-score is an overall measure of the model’s accuracy that combines precision and recall. Specificity (False Positive Rate) is the ratio between the false-negative data that is mistakenly considered positive and all negative data. AUC is defined as the area under the Receiver Operating Characteristic (ROC) Curve.

**Table 5:** The used performance metrics.

Metric	Definition	Formula
Accuracy	The ratio between the correct predictions made by the model and all kinds’ predictions made.	$\frac{TP+TN}{TP+TN+FP+FN}$
Precision	The ratio between the true positive predicted values and full positive predicted values.	$\frac{TP}{TP+FP}$
Recall or Sensitivity	The ratio between the true positive values of prediction and all predicted values.	$\frac{TP}{TP+FN}$
F1-score	Twice the ratio between the multiplication to the summation of precision and recall metrics.	$\frac{2\times TP}{2\times TP+FP+FN}$
Specificity	The ratio between the false-negative data that is mistakenly considered positive and all negative data.	$\frac{FP}{TN+FP}$
AUC	Plotting the cumulative distribution function of the True Positive Rate (TPR) in the y-axis versus the cumulative distribution function of the False Positive Rate (FPR) on the X-axis.	$TPR=\frac{TP}{TP+FN}$ and $FPR=\frac{FP}{FP+TN}$
IoU Coefficient	The ratio between the area of intersection and area of union.	$\frac{TP}{TP+TN+FN}$
Dice Coefficient	Twice the ratio between the true positive predicted values and all other values.	$\frac{2\times TP}{TP+TN+FP+FN}$
Loss	The distance between the true values of the problem and the values predicted by the model.	$l(y,p)=\Sigma_{c=1}^{M}y_{o,c}\times \text{log}p_{o,c}$

The ROC curve is generated by plotting the True Positive (TP) cumulative distribution function in the y-axis versus the False Positive (FP) cumulative distribution function on the X-axis. The AUC is a single-valued metric, and as the AUC value gets higher as the performance of the model increases and easily distinguishes between the different classes. IoU (Intersection over Union) score is considered good when its value is greater than 0.5. The loss is the distance between the true values of the problem and the values predicted by the model. The lower the loss, the better a model unless the model has overfitted to the training data. In the loss formula, *M* is the number of classes, *l* is the loss value, and *p* is the predicted value.

### Results and Discussion

**DenseNet121**: Table 6 reports the DenseNet121 top-1 results after applying 15 iterations of learning and optimization. The table is sorted in ascending order according to the evaluation accuracy. The best achieved results in all iterations for the loss, accuracy, F1, precision, recall, specificity, AUC, sensitivity, IoU, and dice scores were 0.1268, 97.39%, 0.9738, 97.44%, 97.33%, 99.15%, 0.9962, 0.9733, 0.9750, and 0.9783 respectively while the top-1 results, concerning the accuracy, were 0.1268, 97.39%, 0.9738, 97.44%, 97.33%, 99.15%, 0.9962, 0.9733, 0.9750, and 0.9783 respectively. They were reported by the AdaMax parameters optimizer, batch size value of 40, dropout ratio of 0.075, and model learning ratio of 55%. Table 7 reports the correlation between the reported performance metrics and the numeric hyperparameters (i.e. batch size, dropout ratio, and model learn ration).

**Table 6:** DenseNet121 learning and optimization top-1 results.

#	PO*	BS*	DR*	MLR*	Loss	Accuracy	F1	Precision	Recall	Specificity	AUC*	Sensitivity	IoU*	Dice
1	RMSProp	32	0.600	10%	0.2614	89.59%	0.8963	89.96%	89.31%	96.68%	0.9866	0.8931	0.8953	0.9110
2	AdaGrad	32	0.025	25%	0.2771	90.36%	0.9044	90.69%	90.20%	96.91%	0.9854	0.9020	0.9141	0.9249
3	AdaMax	32	0.600	10%	0.2529	90.71%	0.9057	90.93%	90.22%	97.00%	0.9875	0.9022	0.8996	0.9149
4	AdaMax	24	0.550	10%	0.2392	90.82%	0.9066	91.12%	90.22%	97.07%	0.9891	0.9022	0.8977	0.9143
5	NAdam	24	0.025	15%	0.2392	91.56%	0.9160	91.78%	91.43%	97.27%	0.9885	0.9143	0.9111	0.9245
6	AdaMax	48	0.325	20%	0.2810	91.69%	0.9177	91.99%	91.56%	97.34%	0.9847	0.9156	0.9258	0.9345
7	AdaMax	72	0.075	50%	0.2292	91.99%	0.9208	92.29%	91.88%	97.44%	0.9895	0.9188	0.9129	0.9263
8	Adam	16	0.000	15%	0.2108	92.64%	0.9268	92.92%	92.45%	97.65%	0.9909	0.9245	0.9225	0.9344
9	AdaMax	32	0.550	10%	0.2002	92.69%	0.9276	93.21%	92.32%	97.76%	0.9917	0.9232	0.9108	0.9260
10	AdaMax	32	0.550	10%	0.1941	92.76%	0.9272	92.96%	92.49%	97.67%	0.9927	0.9249	0.9129	0.9277
11	Adam	24	0.000	20%	0.1897	93.03%	0.9297	93.42%	92.54%	97.83%	0.9928	0.9254	0.9093	0.9255
12	NAdam	24	0.025	15%	0.2334	93.18%	0.9321	93.34%	93.09%	97.79%	0.9891	0.9309	0.9420	0.9487
13	Adam	64	0.025	55%	0.1846	94.61%	0.9460	94.76%	94.45%	98.26%	0.9920	0.9445	0.9500	0.9562
14	AdaMax	40	0.075	55%	0.1494	96.04%	0.9604	96.08%	96.01%	98.69%	0.9945	0.9601	0.9666	0.9701
15	AdaMax	40	0.075	55%	0.1268	97.39%	0.9738	97.44%	97.33%	99.15%	0.9962	0.9733	0.9750	0.9783

**Note:**

* PO, parameters optimizer; BS, batch size; DR, dropout ratio; MLR, model learn ratio; AUC, area under curve; IoU, intersection over union.

**Table 7:** DenseNet121 model correlation results.

Metric	Batch size	Dropout ratio	Model learn ratio
Loss	−0.1053	0.2908	−0.5941
Accuracy	0.2305	−0.4626	0.7213
F1	0.2410	−0.4763	0.7279
Precision	0.2282	−0.4580	0.7132
Recall	0.2517	−0.4921	0.7397
Specificity	0.2251	−0.4526	0.7095
AUC	0.0421	−0.2492	0.5048
Sensitivity	0.2517	−0.4921	0.7397
IoU Coef	0.2899	−0.5523	0.7699
Dice Coef	0.2781	−0.5439	0.7694

**DenseNet169**: Table 8 reports the DenseNet169 top-1 results after applying 15 iterations of learning and optimization. The table is sorted in ascending order according to the evaluation accuracy. The best achieved results in all iterations for the loss, accuracy, F1, precision, recall, specificity, AUC, sensitivity, IoU, and dice scores were 0.0523, 98.47%, 0.9849, 98.50%, 98.47%, 99.50%, 0.9983, 0.9847, 0.9860, and 0.9879 respectively while the top-1 results, concerning the accuracy, were 0.0523, 98.47%, 0.9849, 98.50%, 98.47%, 99.50%, 0.9983, 0.9847, 0.9860, and 0.9879 respectively. They were reported by the SGD parameters optimizer, batch size value of 88, dropout ratio of 0.025, and model learning ratio of 90%. Table 9 reports the correlation between the reported performance metrics and the numeric hyperparameters (i.e. batch size, dropout ratio, and model learn ration).

**Table 8:** DenseNet169 learning and optimization top-1 results.

#	PO*	BS*	DR*	MLR*	Loss	Accuracy	F1	Precision	Recall	Specificity	AUC*	Sensitivity	IoU*	Dice
1	AdaGrad	96	0.000	100%	0.1524	94.94%	0.9491	95.03%	94.79%	98.35%	0.9943	0.9479	0.9517	0.9580
2	AdaMax	56	0.000	60%	0.1864	94.95%	0.9493	95.04%	94.82%	98.35%	0.9925	0.9482	0.9527	0.9585
3	SGD	88	0.025	90%	0.1349	95.09%	0.9511	95.18%	95.03%	98.40%	0.9951	0.9503	0.9538	0.9604
4	NAdam	72	0.000	70%	0.1410	95.28%	0.9530	95.40%	95.20%	98.47%	0.9948	0.9520	0.9550	0.9613
5	AdaMax	96	0.000	100%	0.1854	95.72%	0.9569	95.71%	95.67%	98.57%	0.9920	0.9567	0.9638	0.9676
6	AdaMax	56	0.000	55%	0.1659	95.85%	0.9594	96.01%	95.86%	98.67%	0.9940	0.9586	0.9643	0.9684
7	NAdam	72	0.000	70%	0.1266	95.91%	0.9587	95.97%	95.77%	98.66%	0.9961	0.9577	0.9568	0.9635
8	NAdam	96	0.000	90%	0.1052	96.05%	0.9605	96.11%	95.99%	98.70%	0.9974	0.9599	0.9561	0.9634
9	RMSProp	64	0.025	70%	0.1698	96.13%	0.9609	96.13%	96.06%	98.71%	0.9943	0.9606	0.9667	0.9704
10	NAdam	88	0.000	85%	0.1120	96.65%	0.9667	96.72%	96.62%	98.91%	0.9961	0.9662	0.9718	0.9751
11	NAdam	88	0.000	85%	0.0975	96.85%	0.9688	96.97%	96.80%	98.99%	0.9962	0.9680	0.9696	0.9737
12	NAdam	56	0.000	55%	0.0854	97.36%	0.9735	97.40%	97.31%	99.13%	0.9968	0.9731	0.9734	0.9774
13	AdaMax	64	0.000	65%	0.0823	98.05%	0.9806	98.07%	98.04%	99.36%	0.9969	0.9804	0.9811	0.9835
14	SGD	88	0.025	90%	0.0617	98.38%	0.9838	98.40%	98.36%	99.47%	0.9981	0.9836	0.9820	0.9847
15	SGD	88	0.025	90%	0.0523	98.47%	0.9849	98.50%	98.47%	99.50%	0.9983	0.9847	0.9860	0.9879

**Note:**

* POO, parameters optimizer; BS, batch size; DR, dropout ratio; MLR, model learn ratio; AUC, area under curve; IoU, intersection over union.

**Table 9:** DenseNet169 model correlation results.

Metric	Batch size	Dropout ratio	Model learn ratio
Loss	−0.2079	−0.2777	−0.1250
Accuracy	0.0105	0.3294	−0.0119
F1	0.0068	0.3264	−0.0180
Precision	0.0037	0.3202	−0.0227
Recall	0.0094	0.3315	−0.0139
Specificity	0.0050	0.3222	−0.0209
AUC	0.1963	0.3047	0.0951
Sensitivity	0.0094	0.3315	−0.0139
IoU Coef	−0.0308	0.3537	−0.0244
Dice Coef	−0.0175	0.3557	−0.0202

**DenseNet201**: Table 10 reports the DenseNet201 top-1 results after applying 15 iterations of learning and optimization. The table is sorted in ascending order according to the evaluation accuracy. The best achieved results in all iterations for the loss, accuracy, F1, precision, recall, specificity, AUC, sensitivity, IoU, and dice scores were 0.1104, 96.69%, 0.9675, 96.90%, 96.61%, 98.97%, 0.9967, 0.9661, 0.9667, and 0.9711 respectively while the top-1 results, concerning the accuracy, were 0.1153, 96.69%, 0.9675, 96.90%, 96.61%, 98.97%, 0.9958, 0.9661, 0.9667, and 0.9711 respectively. They were reported by the SGD parameters optimizer, batch size value of 80, dropout ratio of 0.000, and model learning ratio of 45%. Table 11 reports the correlation between the reported performance metrics and the numeric hyperparameters (i.e. batch size, dropout ratio, and model learn ration).

**Table 10:** DenseNet201 learning and optimization top-1 results.

#	PO*	BS*	DR*	MLR*	Loss	Accuracy	F1	Precision	Recall	Specificity	AUC*	Sensitivity	IoU*	Dice
1	SGD	72	0.000	35%	0.2268	92.58%	0.9268	92.90%	92.46%	97.65%	0.9885	0.9246	0.9275	0.9374
2	RMSProp	56	0.050	50%	2.0370	92.60%	0.9256	92.63%	92.49%	97.55%	0.9812	0.9249	0.9390	0.9451
3	AdaMax	80	0.125	60%	0.2259	93.25%	0.9326	93.37%	93.15%	97.80%	0.9892	0.9315	0.9372	0.9448
4	SGD	80	0.000	45%	0.1912	93.50%	0.9344	93.55%	93.32%	97.86%	0.9925	0.9332	0.9415	0.9488
5	AdaMax	56	0.000	25%	0.1833	93.59%	0.9361	93.76%	93.46%	97.93%	0.9933	0.9346	0.9361	0.9452
6	SGD	88	0.000	40%	0.1874	93.80%	0.9383	93.91%	93.76%	97.97%	0.9928	0.9376	0.9456	0.9521
7	SGD	80	0.000	40%	0.2059	93.93%	0.9397	94.17%	93.78%	98.06%	0.9898	0.9378	0.9411	0.9485
8	AdaMax	56	0.000	25%	0.1776	94.10%	0.9417	94.38%	93.96%	98.13%	0.9931	0.9396	0.9429	0.9508
9	RMSProp	80	0.000	40%	0.2449	94.99%	0.9497	95.00%	94.95%	98.33%	0.9886	0.9495	0.9616	0.9646
10	AdaMax	56	0.000	25%	0.1660	95.02%	0.9504	95.15%	94.93%	98.39%	0.9929	0.9493	0.9526	0.9586
11	SGD	88	0.000	40%	0.1551	95.15%	0.9516	95.24%	95.07%	98.42%	0.9944	0.9507	0.9530	0.9590
12	SGD	88	0.000	50%	0.1193	96.09%	0.9608	96.23%	95.94%	98.75%	0.9960	0.9594	0.9603	0.9660
13	SGD	80	0.000	40%	0.1104	96.14%	0.9615	96.28%	96.02%	98.76%	0.9967	0.9602	0.9595	0.9658
14	RMSProp	72	0.000	40%	0.1356	96.27%	0.9630	96.37%	96.24%	98.79%	0.9952	0.9624	0.9653	0.9696
15	SGD	80	0.000	45%	0.1153	96.69%	0.9675	96.90%	96.61%	98.97%	0.9958	0.9661	0.9667	0.9711

**Note:**

* PO, parameters optimizer; BS, batch size; DR, dropout ratio; MLR, model learn ratio; AUC, area under curve; IoU, intersection over union.

**Table 11:** DenseNet201 model correlation results.

Metric	Batch size	Dropout ratio	Model learn ratio
Loss	−0.4154	0.3397	0.2799
Accuracy	0.3403	−0.3941	−0.0038
F1	0.3359	−0.3995	−0.0169
Precision	0.3299	−0.4090	−0.0302
Recall	0.3415	−0.3895	−0.0032
Specificity	0.3294	−0.4085	−0.0301
AUC	0.3475	−0.4665	−0.2434
Sensitivity	0.3415	−0.3895	−0.0032
IoU Coef	0.3457	−0.3346	0.1004
Dice Coef	0.3447	−0.3607	0.0696

**Xception**: Table 12 reports the Xception top-1 results after applying 15 iterations of learning and optimization. The table is sorted in ascending order according to the evaluation accuracy. The best achieved results in all iterations for the loss, accuracy, F1, precision, recall, specificity, AUC, sensitivity, IoU, and dice scores were 0.0861, 97.78%, 0.9783, 97.90%, 97.76%, 99.30%, 0.9979, 0.9776, 0.9750, and 0.9789 respectively while the top-1 results, concerning the accuracy, were 0.0888, 97.78%, 0.9783, 97.90%, 97.76%, 99.30%, 0.9961, 0.9776, 0.9750, and 0.9789 respectively. They were reported by the RMSProp parameters optimizer, batch size value of 80, dropout ratio of 0.525, and model learning ratio of 70%. Table 13 reports the correlation between the reported performance metrics and the numeric hyperparameters (i.e. batch size, dropout ratio, and model learn ration).

**Table 12:** Xception learning and optimization top-1 results.

#	PO*	BS*	DR*	MLR*	Loss	Accuracy	F1	Precision	Recall	Specificity	AUC*	Sensitivity	IoU*	Dice
1	RMSProp	80	0.525	70%	0.1574	95.12%	0.9516	95.28%	95.03%	98.43%	0.9933	0.9503	0.9560	0.9617
2	NAdam	24	0.100	15%	0.1757	95.26%	0.9530	95.37%	95.23%	98.46%	0.9925	0.9523	0.9594	0.9634
3	RMSProp	96	0.600	75%	0.2901	95.50%	0.9549	95.51%	95.47%	98.50%	0.9894	0.9547	0.9638	0.9669
4	RMSProp	88	0.550	70%	0.1571	95.69%	0.9571	95.84%	95.58%	98.62%	0.9946	0.9558	0.9630	0.9671
5	NAdam	24	0.125	20%	0.1279	95.83%	0.9572	95.82%	95.61%	98.61%	0.9961	0.9561	0.9536	0.9610
6	AdaMax	56	0.375	55%	0.1394	95.89%	0.9582	95.95%	95.70%	98.65%	0.9943	0.9570	0.9487	0.9573
7	NAdam	24	0.150	25%	0.1115	96.06%	0.9606	96.11%	96.01%	98.71%	0.9971	0.9601	0.9565	0.9636
8	NAdam	24	0.100	15%	0.1138	96.13%	0.9615	96.30%	96.00%	98.77%	0.9969	0.9600	0.9545	0.9620
9	NAdam	24	0.100	15%	0.1097	96.17%	0.9616	96.22%	96.10%	98.74%	0.9968	0.9610	0.9558	0.9631
10	NAdam	24	0.100	15%	0.0957	96.31%	0.9628	96.37%	96.19%	98.79%	0.9979	0.9619	0.9573	0.9647
11	NAdam	24	0.100	15%	0.1031	96.41%	0.9640	96.48%	96.32%	98.83%	0.9975	0.9632	0.9567	0.9641
12	RMSProp	96	0.600	75%	0.1374	96.79%	0.9676	96.78%	96.74%	98.93%	0.9940	0.9674	0.9728	0.9756
13	NAdam	24	0.100	15%	0.0861	96.99%	0.9699	97.07%	96.91%	99.03%	0.9979	0.9691	0.9627	0.9692
14	RMSProp	88	0.550	70%	0.1618	97.12%	0.9710	97.15%	97.05%	99.05%	0.9967	0.9705	0.9723	0.9757
15	RMSProp	80	0.525	70%	0.0888	97.78%	0.9783	97.90%	97.76%	99.30%	0.9961	0.9776	0.9750	0.9789

**Note:**

* PO, parameters optimizer; BS, batch size; DR, dropout ratio; MLR, model learn ratio; AUC, area under curve; IoU, intersection over union.

**Table 13:** Xception model correlation results.

Metric	Batch size	Dropout ratio	Model learn ratio
Loss	0.5463	0.5275	0.5068
Accuracy	0.1073	0.1006	0.0943
F1	0.1169	0.1094	0.1023
Precision	0.1042	0.0979	0.0923
Recall	0.1288	0.1202	0.1117
Specificity	0.1005	0.0943	0.0888
AUC	−0.5791	−0.5741	−0.5661
Sensitivity	0.1288	0.1202	0.1117
IoU Coef	0.6394	0.6098	0.5760
Dice Coef	0.5584	0.5324	0.5025

**MobileNet**: Table 14 reports the MobileNet top-1 results after applying 15 iterations of learning and optimization. The table is sorted in ascending order according to the evaluation accuracy. The best achieved results in all iterations for the loss, accuracy, F1, precision, recall, specificity, AUC, sensitivity, IoU, and dice scores were 0.1247, 96.40%, 0.9636, 96.39%, 96.33%, 98.80%, 0.9952, 0.9633, 0.9664, and 0.9706 respectively while the top-1 results, concerning the accuracy, were 0.1247, 96.40%, 0.9636, 96.39%, 96.33%, 98.80%, 0.9952, 0.9633, 0.9664, and 0.9706 respectively. They were reported by the NAdam parameters optimizer, batch size value of 56, dropout ratio of 0.050, and model learning ratio of 25%. Table 15 reports the correlation between the reported performance metrics and the numeric hyperparameters (i.e. batch size, dropout ratio, and model learn ration).

**Table 14:** MobileNet learning and optimization top-1 results.

#	PO*	BS*	DR*	MLR*	Loss	Accuracy	F1	Precision	Recall	Specificity	AUC*	Sensitivity	IoU*	Dice
1	SGD	96	0.325	100%	0.3538	87.74%	0.8779	88.32%	87.29%	96.15%	0.9790	0.8729	0.8864	0.9013
2	Adam	8	0.200	30%	0.2981	87.75%	0.8782	88.77%	86.91%	96.33%	0.9824	0.8691	0.8720	0.8921
3	NAdam	40	0.050	20%	0.3393	89.47%	0.8948	89.64%	89.33%	96.56%	0.9806	0.8933	0.9121	0.9215
4	SGD	96	0.325	100%	0.3017	89.97%	0.9002	90.41%	89.64%	96.83%	0.9823	0.8964	0.9031	0.9163
5	NAdam	24	0.100	15%	0.2593	90.09%	0.9011	90.59%	89.66%	96.90%	0.9870	0.8966	0.8945	0.9105
6	Adam	32	0.025	15%	0.2775	91.44%	0.9138	91.63%	91.13%	97.22%	0.9864	0.9113	0.9177	0.9287
7	NAdam	48	0.050	25%	0.2553	92.34%	0.9230	92.60%	92.01%	97.55%	0.9861	0.9201	0.9297	0.9383
8	AdaMax	24	0.450	45%	0.2351	92.39%	0.9239	92.63%	92.16%	97.56%	0.9890	0.9216	0.9238	0.9345
9	RMSProp	24	0.475	45%	0.2552	92.78%	0.9282	93.04%	92.62%	97.69%	0.9878	0.9262	0.9289	0.9387
10	NAdam	72	0.075	35%	0.2226	93.43%	0.9342	93.56%	93.28%	97.86%	0.9885	0.9328	0.9374	0.9456
11	SGD	32	0.600	60%	0.2281	93.68%	0.9361	93.67%	93.55%	97.89%	0.9896	0.9355	0.9453	0.9513
12	Adam	8	0.175	30%	0.1777	94.35%	0.9423	94.63%	93.84%	98.23%	0.9934	0.9384	0.9187	0.9334
13	SGD	16	0.600	50%	0.1740	94.51%	0.9446	94.66%	94.26%	98.23%	0.9929	0.9426	0.9300	0.9418
14	AdaMax	24	0.450	45%	0.1444	95.85%	0.9576	96.06%	95.46%	98.69%	0.9938	0.9546	0.9483	0.9567
15	NAdam	56	0.050	25%	0.1247	96.40%	0.9636	96.39%	96.33%	98.80%	0.9952	0.9633	0.9664	0.9706

**Note:**

* PO, parameters optimizer; BS, batch size; DR, dropout ratio; MLR, model learn ratio; AUC, area under curve; IoU, intersection over union.

**Table 15:** MobileNet model correlation results.

Metric	Batch size	Dropout ratio	Model learn ratio
Loss	0.3770	−0.1914	0.2906
Accuracy	−0.2703	0.2201	−0.2438
F1	−0.2666	0.2226	−0.2407
Precision	−0.2938	0.2259	−0.2452
Recall	−0.2424	0.2191	−0.2363
Specificity	−0.2990	0.2258	−0.2463
AUC	−0.4568	0.2247	−0.3325
Sensitivity	−0.2424	0.2191	−0.2363
IoU Coef	−0.0551	0.1504	−0.1983
Dice Coef	−0.0981	0.1654	−0.2108

**MobileNetV2**: Table 16 reports the MobileNetV2 top-1 results after applying 15 iterations of learning and optimization. The table is sorted in ascending order according to the evaluation accuracy. The best achieved results in all iterations for the loss, accuracy, F1, precision, recall, specificity, AUC, sensitivity, IoU, and dice scores were 0.6045, 81.54%, 0.8158, 81.69%, 81.48%, 93.91%, 0.9417, 0.8148, 0.8635, and 0.8728 respectively while the top-1 results, concerning the accuracy, were 2.5140, 81.54%, 0.8158, 81.69%, 81.48%, 93.91%, 0.9352, 0.8148, 0.8635, and 0.8728 respectively. They were reported by the RMSProp parameters optimizer, batch size value of 16, dropout ratio of 0.425, and model learning ratio of 55%. Table 17 reports the correlation between the reported performance metrics and the numeric hyperparameters (i.e. batch size, dropout ratio, and model learn ration).

**Table 16:** MobileNetV2 learning and optimization top-1 results.

#	PO*	BS*	DR*	MLR*	Loss	Accuracy	F1	Precision	Recall	Specificity	AUC*	Sensitivity	IoU*	Dice
1	Adam	8	0.000	0%	0.6198	75.70%	0.7524	78.53%	72.31%	93.42%	0.9376	0.7231	0.7659	0.7967
2	Adam	8	0.000	0%	0.6198	75.70%	0.7524	78.53%	72.31%	93.42%	0.9376	0.7231	0.7659	0.7967
3	Adam	8	0.000	0%	0.6110	75.87%	0.7505	78.32%	72.13%	93.34%	0.9399	0.7213	0.7722	0.8016
4	Adam	8	0.000	0%	0.6110	75.87%	0.7505	78.32%	72.13%	93.34%	0.9399	0.7213	0.7722	0.8016
5	Adam	8	0.000	0%	0.6290	76.37%	0.7595	79.11%	73.14%	93.57%	0.9370	0.7314	0.7723	0.8015
6	Adam	8	0.000	0%	0.6290	76.37%	0.7595	79.11%	73.14%	93.57%	0.9370	0.7314	0.7723	0.8015
7	Adam	8	0.000	0%	0.6100	76.69%	0.7617	78.93%	73.67%	93.44%	0.9398	0.7367	0.7774	0.8058
8	Adam	8	0.000	0%	0.6068	76.70%	0.7625	79.13%	73.66%	93.52%	0.9399	0.7366	0.7728	0.8031
9	Adam	8	0.000	0%	0.6045	77.02%	0.7656	79.36%	74.03%	93.58%	0.9417	0.7403	0.7779	0.8071
10	Adam	8	0.000	0%	0.6045	77.02%	0.7656	79.36%	74.03%	93.58%	0.9417	0.7403	0.7779	0.8071
11	Adam	8	0.000	0%	0.6045	77.02%	0.7656	79.36%	74.03%	93.58%	0.9417	0.7403	0.7779	0.8071
12	Adam	8	0.000	0%	0.6045	77.02%	0.7656	79.36%	74.03%	93.58%	0.9417	0.7403	0.7779	0.8071
13	Adam	8	0.000	0%	0.6045	77.02%	0.7656	79.36%	74.03%	93.58%	0.9417	0.7403	0.7779	0.8071
14	Adam	8	0.000	0%	0.6045	77.02%	0.7656	79.36%	74.03%	93.58%	0.9417	0.7403	0.7779	0.8071
15	RMSProp	16	0.425	55%	2.5140	81.54%	0.8158	81.69%	81.48%	93.91%	0.9352	0.8148	0.8635	0.8728

**Note:**

* PO, parameters optimizer; BS, batch size; DR, dropout ratio; MLR, model learn ratio; AUC, area under curve; IoU, intersection over union.

**Table 17:** MobileNetV2 model correlation results.

Metric	Batch Size	Dropout Ratio	Model Learn Ratio
Loss	0.9998	0.9998	0.9998
Accuracy	0.9277	0.9277	0.9277
F1	0.9241	0.9241	0.9241
Precision	0.8682	0.8682	0.8682
Recall	0.9397	0.9397	0.9397
Specificity	0.7533	0.7533	0.7533
AUC	−0.5546	−0.5546	−0.5546
Sensitivity	0.9397	0.9397	0.9397
IoU Coef	0.9838	0.9838	0.9838
Dice Coef	0.9793	0.9793	0.9793

**MobileNetV3Small**: Table 18 reports the MobileNetV3Small top-1 results after applying 15 iterations of learning and optimization. The table is sorted in ascending order according to the evaluation accuracy. The best achieved results in all iterations for the loss, accuracy, F1, precision, recall, specificity, AUC, sensitivity, IoU, and dice scores were 0.7490, 80.06%, 0.7995, 80.50%, 79.43%, 97.76%, 0.9380, 0.7943, 0.8183, and 0.8381 respectively while the top-1 results, concerning the accuracy, were 27.860, 80.06%, 0.7995, 80.50%, 79.43%, 93.59%, 0.9270, 0.7943, 0.8159, and 0.8365 respectively. They were reported by the RMSProp parameters optimizer, batch size value of 8, dropout ratio of 0.075, and model learning ratio of 100%. Table 19 reports the correlation between the reported performance metrics and the numeric hyperparameters (i.e. batch size, dropout ratio, and model learn ration).

**Table 18:** MobileNetV3Small learning and optimization top-1 results.

#	PO*	BS*	DR*	MLR*	Loss	Accuracy	F1	Precision	Recall	Specificity	AUC*	Sensitivity	IoU*	Dice
1	Adam	8	0.000	0%	0.9894	55.61%	0.4983	71.14%	38.65%	94.75%	0.8318	0.3865	0.5649	0.6192
2	Adam	8	0.000	0%	0.9894	55.61%	0.4983	71.14%	38.65%	94.75%	0.8318	0.3865	0.5649	0.6192
3	AdaDelta	40	0.075	55%	0.9877	58.12%	0.5627	63.96%	50.45%	90.53%	0.8370	0.5045	0.6332	0.6749
4	Adam	8	0.000	0%	1.0320	58.49%	0.3180	75.81%	20.50%	97.76%	0.8247	0.2050	0.5357	0.5922
5	Adam	8	0.000	0%	1.0320	58.49%	0.3180	75.81%	20.50%	97.76%	0.8247	0.2050	0.5357	0.5922
6	Adam	8	0.000	0%	1.0320	58.49%	0.3180	75.81%	20.50%	97.76%	0.8247	0.2050	0.5357	0.5922
7	NAdam	8	0.000	25%	0.9177	60.73%	0.5948	64.47%	55.34%	89.82%	0.8647	0.5534	0.6570	0.6989
8	Adam	8	0.000	0%	0.9881	61.23%	0.4416	70.65%	32.55%	95.50%	0.8416	0.3255	0.5613	0.6159
9	Adam	8	0.000	0%	0.9881	61.23%	0.4416	70.65%	32.55%	95.50%	0.8416	0.3255	0.5613	0.6159
10	Adam	8	0.000	0%	0.9736	62.57%	0.4341	73.52%	31.22%	96.25%	0.8492	0.3122	0.5568	0.6131
11	Adam	8	0.000	0%	0.9736	62.57%	0.4341	73.52%	31.22%	96.25%	0.8492	0.3122	0.5568	0.6131
12	AdaDelta	40	0.075	55%	0.9041	63.23%	0.6162	68.65%	56.10%	91.44%	0.8636	0.5610	0.6605	0.7007
13	AdaGrad	8	0.025	40%	0.7490	70.61%	0.6714	78.95%	58.66%	94.81%	0.9046	0.5866	0.6709	0.7157
14	RMSProp	8	0.075	100%	1.1210	79.15%	0.7897	79.65%	78.32%	93.33%	0.9380	0.7832	0.8183	0.8381
15	RMSProp	8	0.075	100%	27.860	80.06%	0.7995	80.50%	79.43%	93.59%	0.9270	0.7943	0.8159	0.8365

**Note:**

* PO, parameters optimizer; BS, batch size; DR, dropout ratio; MLR, model learn ratio; AUC, area under curve; IoU, intersection over union.

**Table 19:** MobileNetV3Small model correlation results.

Metric	Batch size	Dropout ratio	Model learn ratio
Loss	−0.1066	0.4354	0.5648
Accuracy	−0.1279	0.6368	0.8362
F1	0.1918	0.7671	0.8984
Precision	−0.5384	0.0936	0.2804
Recall	0.2159	0.7967	0.9284
Specificity	−0.5904	−0.6059	−0.5832
AUC	−0.0728	0.6555	0.8645
Sensitivity	0.2159	0.7967	0.9284
IoU Coef	0.1355	0.8106	0.9677
Dice Coef	0.1248	0.8031	0.9639

**MobileNetV3Large**: Table 20 reports the MobileNetV3Large top-1 results after applying 15 iterations of learning and optimization. The table is sorted in ascending order according to the evaluation accuracy. The best achieved results in all iterations for the loss, accuracy, F1, precision, recall, specificity, AUC, sensitivity, IoU, and dice scores were 0.5192, 79.57%, 0.7851, 83.15%, 74.50%, 95.12%, 0.9531, 0.7450, 0.7550, and 0.7931 respectively while the top-1 results, concerning the accuracy, were 0.5192, 79.57%, 0.7851, 83.15%, 74.50%, 94.97%, 0.9531, 0.7450, 0.7550, and 0.7931 respectively. They were reported by the AdaMax parameters optimizer, batch size value of 16, dropout ratio of 0.175, and model learning ratio of 20%. Table 21 reports the correlation between the reported performance metrics and the numeric hyperparameters (i.e. batch size, dropout ratio, and model learn ration).

**Table 20:** MobileNetV3Large learning and optimization top-1 results.

#	PO*	BS*	DR*	MLR*	Loss	Accuracy	F1	Precision	Recall	Specificity	AUC*	Sensitivity	IoU*	Dice
1	Adam	8	0.000	0%	0.8226	68.66%	0.6448	77.23%	55.62%	94.52%	0.8866	0.5562	0.6415	0.6907
2	Adam	8	0.000	0%	0.8226	68.66%	0.6448	77.23%	55.62%	94.52%	0.8866	0.5562	0.6415	0.6907
3	Adam	8	0.000	0%	0.7898	70.73%	0.6601	79.48%	56.74%	95.12%	0.8971	0.5674	0.6457	0.6953
4	Adam	8	0.000	0%	0.7898	70.73%	0.6601	79.48%	56.74%	95.12%	0.8971	0.5674	0.6457	0.6953
5	Adam	8	0.000	0%	0.7898	70.73%	0.6601	79.48%	56.74%	95.12%	0.8971	0.5674	0.6457	0.6953
6	Adam	8	0.000	0%	0.8042	71.07%	0.6517	77.69%	56.44%	94.60%	0.8927	0.5644	0.6352	0.6870
7	Adam	8	0.000	0%	0.8042	71.07%	0.6517	77.69%	56.44%	94.60%	0.8927	0.5644	0.6352	0.6870
8	Adam	8	0.000	0%	0.8042	71.07%	0.6517	77.69%	56.44%	94.60%	0.8927	0.5644	0.6352	0.6870
9	Adam	8	0.000	0%	0.8042	71.07%	0.6517	77.69%	56.44%	94.60%	0.8927	0.5644	0.6352	0.6870
10	AdaDelta	8	0.125	15%	0.6713	71.79%	0.7056	76.87%	65.40%	93.45%	0.9212	0.6540	0.6968	0.7403
11	AdaDelta	8	0.125	15%	0.6632	71.90%	0.7065	77.63%	65.00%	93.76%	0.9230	0.6500	0.7059	0.7474
12	AdaDelta	8	0.100	15%	0.7113	71.99%	0.7028	77.77%	64.30%	93.87%	0.9117	0.6430	0.6955	0.7374
13	AdaDelta	8	0.125	15%	0.6725	72.91%	0.7204	78.43%	66.77%	93.88%	0.9214	0.6677	0.7166	0.7553
14	AdaDelta	8	0.125	15%	0.6711	73.09%	0.7202	78.62%	66.62%	93.97%	0.9217	0.6662	0.7163	0.7552
15	AdaMax	16	0.175	20%	0.5192	79.57%	0.7851	83.15%	74.50%	94.97%	0.9531	0.7450	0.7550	0.7931

**Note:**

* PO, parameters optimizer; BS, batch size; DR, dropout ratio; MLR, model learn ratio; AUC, area under curve; IoU, intersection over union.

**Table 21:** MobileNetV3Large model correlation results.

Metric	Batch size	Dropout ratio	Model learn ratio
Loss	−0.7073	−0.9595	−0.9387
Accuracy	0.8701	0.7588	0.7283
F1	0.7091	0.9543	0.9381
Precision	0.8393	0.3440	0.3038
Recall	0.6494	0.9804	0.9681
Specificity	0.2680	−0.6046	−0.6364
AUC	0.6954	0.9539	0.9323
Sensitivity	0.6494	0.9804	0.9681
IoU Coef	0.5888	0.9843	0.9745
Dice Coef	0.6102	0.9836	0.9723

**EfficientNetB0**: Table 22 reports the EfficientNetB0 top-1 results after applying 15 iterations of learning and optimization. The table is sorted in ascending order according to the evaluation accuracy. The best achieved results in all iterations for the loss, accuracy, F1, precision, recall, specificity, AUC, sensitivity, IoU, and dice scores were 1.0970, 51.23%, 0.5070, 92.71%, 48.72%, 100.0%, 0.8072, 0.4872, 0.6478, and 0.6724 respectively while the top-1 results, concerning the accuracy, were 1.3980, 51.23%, 0.5070, 52.92%, 48.72%, 85.55%, 0.8003, 0.4872, 0.6478, and 0.6724 respectively. They were reported by the RMSProp parameters optimizer, batch size value of 64, dropout ratio of 0.375, and model learning ratio of 80%. Table 23 reports the correlation between the reported performance metrics and the numeric hyperparameters (i.e. batch size, dropout ratio, and model learn ration).

**Table 22:** EfficientNetB0 learning and optimization top-1 results.

#	PO*	BS*	DR*	MLR*	Loss	Accuracy	F1	Precision	Recall	Specificity	AUC*	Sensitivity	IoU*	Dice
1	Ftrl	96	0.600	100%	1.3860	27.61%	0.0000	0.00%	0.00 %	100.00%	0.5174	0.0000	0.4546	0.5001
2	NAdam	24	0.100	15%	1.4030	27.61%	0.0000	0.00%	0.00 %	100.00%	0.5263	0.0000	0.4578	0.5029
3	Adam	8	0.025	5%	1.3850	27.61%	0.0000	0.00%	0.00 %	100.00%	0.5283	0.0000	0.4551	0.5007
4	AdaMax	16	0.250	20%	1.4160	27.61%	0.1125	63.48%	6.30%	98.86%	0.5479	0.0630	0.4719	0.5173
5	SGD	88	0.275	100%	1.8270	27.77%	0.2868	33.40%	25.25%	83.21%	0.6204	0.2525	0.5341	0.5557
6	AdaMax	64	0.400	65%	1.2800	27.97%	0.2946	33.80%	26.22%	82.82%	0.7180	0.2622	0.5146	0.5603
7	SGD	88	0.375	95%	1.3910	28.90%	0.0230	25.92%	1.22%	99.34%	0.5543	0.0122	0.4625	0.5081
8	AdaDelta	48	0.150	50%	1.3880	31.70%	0.2163	39.42%	15.12%	92.22%	0.6085	0.1512	0.4891	0.5326
9	AdaGrad	48	0.050	50%	2.7130	36.25%	0.3615	36.23%	36.07%	78.83%	0.6923	0.3607	0.5642	0.5772
10	AdaMax	16	0.275	20%	1.2570	36.43%	0.1971	92.71%	11.26%	99.81%	0.6712	0.1126	0.4865	0.5354
11	AdaGrad	88	0.400	60%	1.2100	38.65%	0.3226	41.50%	26.59%	87.51%	0.7317	0.2659	0.5088	0.5591
12	AdaGrad	48	0.150	80%	1.1590	45.03%	0.3849	51.30%	31.05%	90.15%	0.7643	0.3105	0.5574	0.6049
13	AdaMax	24	0.400	30%	4.4170	45.29%	0.4335	45.38%	41.55%	83.31%	0.5967	0.4155	0.5144	0.5513
14	SGD	88	0.425	100%	1.0970	50.95%	0.4904	54.48%	44.71%	87.54%	0.8072	0.4471	0.6118	0.6525
15	RMSProp	64	0.375	80%	1.3980	51.23%	0.5070	52.92%	48.72%	85.55%	0.8003	0.4872	0.6478	0.6724

**Note:**

* PO, parameters optimizer; BS, batch size; DR, dropout ratio; MLR, model learn ratio; AUC, area under curve; IoU, intersection over union.

**Table 23:** EfficientNetB0 model correlation results.

Metric	Batch size	Dropout ratio	Model learn ratio
Loss	−0.2555	−0.0024	−0.2248
Accuracy	0.1105	0.1989	0.2437
F1	0.2052	0.1662	0.3176
Precision	−0.1674	0.0925	−0.0458
Recall	0.2264	0.1731	0.3315
Specificity	−0.2941	−0.0617	−0.3210
AUC	0.3096	0.1485	0.3918
Sensitivity	0.2264	0.1731	0.3315
IoU Coef	0.2958	0.1116	0.4449
Dice Coef	0.2969	0.1630	0.4439

**ResNet50V2**: Table 24 reports the ResNet50V2 top-1 results after applying 15 iterations of learning and optimization. The table is sorted in ascending order according to the evaluation accuracy. The best achieved results in all iterations for the loss, accuracy, F1, precision, recall, specificity, AUC, sensitivity, IoU, and dice scores were 0.0792, 97.46%, 0.9737, 97.60%, 97.14%, 99.20%, 0.9984, 0.9714, 0.9721, and 0.9750 respectively while the top-1 results, concerning the accuracy, were 0.0792, 97.46%, 0.9737, 97.60%, 97.14%, 99.20%, 0.9984, 0.9714, 0.9635, and 0.9703 respectively. They were reported by the AdaGrad parameters optimizer, batch size value of 16, dropout ratio of 0.100, and model learning ratio of 30%. Table 25 reports the correlation between the reported performance metrics and the numeric hyperparameters (i.e. batch size, dropout ratio, and model learn ration).

**Table 24:** ResNet50V2 learning and optimization top-1 results.

#	PO*	BS*	DR*	MLR*	Loss	Accuracy	F1	Precision	Recall	Specificity	AUC*	Sensitivity	IoU*	Dice
1	RMSProp	80	0.500	25%	0.3382	89.52%	0.8941	89.78%	89.06%	96.62%	0.9797	0.8906	0.8969	0.9114
2	AdaMax	88	0.475	10%	0.3767	89.53%	0.8959	89.89%	89.30%	96.65%	0.9779	0.8930	0.9077	0.9180
3	AdaGrad	16	0.100	30%	0.2124	91.81%	0.9188	92.36%	91.43%	97.48%	0.9911	0.9143	0.9055	0.9213
4	NAdam	8	0.050	15%	0.2144	91.84%	0.9202	92.39%	91.66%	97.48%	0.9903	0.9166	0.9066	0.9222
5	AdaGrad	16	0.125	35%	0.2326	92.09%	0.9217	92.42%	91.93%	97.49%	0.9891	0.9193	0.9266	0.9362
6	NAdam	8	0.050	15%	0.2144	92.96%	0.9302	93.13%	92.92%	97.71%	0.9906	0.9292	0.9335	0.9423
7	NAdam	8	0.050	15%	0.1843	93.33%	0.9327	93.56%	92.99%	97.87%	0.9933	0.9299	0.9304	0.9407
8	NAdam	8	0.050	15%	0.2119	93.56%	0.9354	93.62%	93.46%	97.88%	0.9905	0.9346	0.9345	0.9436
9	AdaMax	72	0.450	25%	0.2266	94.23%	0.9427	94.38%	94.17%	98.13%	0.9885	0.9417	0.9513	0.9565
10	AdaGrad	16	0.100	30%	0.1717	94.35%	0.9434	94.74%	93.96%	98.26%	0.9937	0.9396	0.9304	0.9419
11	AdaMax	72	0.450	25%	0.1161	96.42%	0.9641	96.50%	96.32%	98.84%	0.9964	0.9632	0.9632	0.9684
12	AdaGrad	16	0.100	30%	0.1204	96.83%	0.9678	96.87%	96.69%	98.96%	0.9956	0.9669	0.9721	0.9750
13	AdaGrad	16	0.100	30%	0.0868	96.96%	0.9700	97.14%	96.87%	99.05%	0.9978	0.9687	0.9637	0.9700
14	AdaGrad	16	0.100	30%	0.0920	97.13%	0.9716	97.31%	97.01%	99.11%	0.9977	0.9701	0.9600	0.9671
15	AdaGrad	16	0.100	30%	0.0792	97.46%	0.9737	97.60%	97.14%	99.20%	0.9984	0.9714	0.9635	0.9703

**Note:**

* PO, parameters optimizer; BS, batch size; DR, dropout ratio; MLR, model learn ratio; AUC, area under curve; IoU, intersection over union.

**Table 25:** ResNet50V2 model correlation results.

Metric	Batch size	Dropout ratio	Model learn ratio
Loss	0.5448	0.5166	−0.5133
Accuracy	−0.3539	−0.3319	0.4854
F1	−0.3611	−0.3395	0.4834
Precision	−0.3719	−0.3503	0.4991
Recall	−0.3509	−0.3291	0.4691
Specificity	−0.3721	−0.3505	0.5021
AUC	−0.6208	−0.5935	0.4764
Sensitivity	−0.3509	−0.3291	0.4691
IoU Coef	−0.1844	−0.1642	0.4113
Dice Coef	−0.2259	−0.2043	0.4341

**ResNet101V2**: Table 26 reports the ResNet101V2 top-1 results after applying 15 iterations of learning and optimization. The table is sorted in ascending order according to the evaluation accuracy. The best achieved results in all iterations for the loss, accuracy, F1, precision, recall, specificity, AUC, sensitivity, IoU, and dice scores were 0.0673, 98.47%, 0.9845, 98.45%, 98.45%, 99.48%, 0.9984, 0.9845, 0.9862, and 0.9878 respectively while the top-1 results, concerning the accuracy, were 0.0673, 98.47%, 0.9845, 98.45%, 98.45%, 99.48%, 0.9972, 0.9845, 0.9862, and 0.9878 respectively. They were reported by the Adam parameters optimizer, batch size value of 56, dropout ratio of 0.075, and model learning ratio of 30%. Table 27 reports the correlation between the reported performance metrics and the numeric hyperparameters (i.e. batch size, dropout ratio, and model learn ration).

**Table 26:** ResNet101V2 learning and optimization top-1 results.

#	PO*	BS*	DR*	MLR*	Loss	Accuracy	F1	Precision	Recall	Specificity	AUC*	Sensitivity	IoU*	Dice
1	Adam	32	0.025	15%	0.1214	96.23%	0.9619	96.25%	96.14%	98.75%	0.9949	0.9614	0.9671	0.9711
2	Adam	32	0.050	15%	0.1114	96.34%	0.9626	96.42%	96.10%	98.81%	0.9966	0.9610	0.9514	0.9600
3	Adam	96	0.175	65%	0.1203	96.58%	0.9659	96.66%	96.53%	98.89%	0.9955	0.9653	0.9653	0.9700
4	Adam	32	0.025	15%	0.0924	96.81%	0.9681	96.86%	96.77%	98.95%	0.9972	0.9677	0.9658	0.9712
5	Adam	40	0.050	20%	0.1036	96.95%	0.9692	96.96%	96.88%	98.99%	0.9965	0.9688	0.9701	0.9741
6	Adam	56	0.075	30%	0.0928	97.21%	0.9725	97.33%	97.17%	99.11%	0.9967	0.9717	0.9658	0.9713
7	Adam	96	0.175	65%	0.1150	97.33%	0.9729	97.32%	97.26%	99.11%	0.9944	0.9726	0.9763	0.9789
8	Adam	40	0.050	20%	0.1052	97.40%	0.9743	97.48%	97.38%	99.16%	0.9955	0.9738	0.9769	0.9796
9	Adam	40	0.050	20%	0.0882	97.52%	0.9743	97.57%	97.30%	99.19%	0.9969	0.9730	0.9669	0.9727
10	Adam	56	0.075	30%	0.0823	97.54%	0.9752	97.53%	97.51%	99.18%	0.9968	0.9751	0.9765	0.9797
11	Adam	96	0.125	45%	0.0788	97.57%	0.9756	97.74%	97.39%	99.25%	0.9977	0.9739	0.9676	0.9733
12	Adam	56	0.075	30%	0.0762	97.57%	0.9758	97.63%	97.53%	99.21%	0.9978	0.9753	0.9741	0.9781
13	Adam	32	0.025	15%	0.0706	97.61%	0.9765	97.69%	97.61%	99.23%	0.9984	0.9761	0.9704	0.9757
14	Adam	56	0.075	30%	0.0673	97.76%	0.9774	97.76%	97.72%	99.25%	0.9984	0.9772	0.9750	0.9790
15	Adam	56	0.075	30%	0.0673	98.47%	0.9845	98.45%	98.45%	99.48%	0.9972	0.9845	0.9862	0.9878

**Note:**

* PO, parameters optimizer; BS, batch size; DR, dropout ratio; MLR, model learn ratio; AUC, area under curve; IoU, intersection over union.

**Table 27:** ResNet101V2 model correlation results.

Metric	Batch size	Dropout ratio	Model learn ratio
Loss	0.0858	0.2366	0.2198
Accuracy	0.1830	0.0798	0.0957
F1	0.1872	0.0804	0.1002
Precision	0.2013	0.0872	0.1000
Recall	0.1745	0.0743	0.1008
Specificity	0.2080	0.0941	0.1061
AUC	−0.2223	−0.3566	−0.3593
Sensitivity	0.1745	0.0743	0.1008
IoU Coef	0.2010	0.1405	0.1986
Dice Coef	0.1876	0.1170	0.1720

**ResNet152V2**: Table 28 reports the ResNet152V2 top-1 results after applying 15 iterations of learning and optimization. The table is sorted in ascending order according to the evaluation accuracy. The best achieved results in all iterations for the loss, accuracy, F1, precision, recall, specificity, AUC, sensitivity, IoU, and dice scores were 0.0935, 96.92%, 0.9694, 97.02%, 96.86%, 99.01%, 0.9972, 0.9686, 0.9679, and 0.9725 respectively while the top-1 results, concerning the accuracy, were 0.1008, 96.92%, 0.9694, 97.02%, 96.86%, 99.01%, 0.9967, 0.9686, 0.9663, and 0.9713 respectively. They were reported by the NAdam parameters optimizer, batch size value of 96, dropout ratio of 0.075, and model learning ratio of 50%. Table 29 reports the correlation between the reported performance metrics and the numeric hyperparameters (i.e. batch size, dropout ratio, and model learn ration).

**Table 28:** ResNet152V2 learning and optimization top-1 results.

#	PO*	BS*	DR*	MLR*	Loss	Accuracy	F1	Precision	Recall	Specificity	AUC*	Sensitivity	IoU*	Dice
1	SGD	32	0.400	35%	0.2944	88.74%	0.8865	89.23%	88.09%	96.46%	0.9838	0.8809	0.8837	0.9009
2	NAdam	64	0.050	45%	0.2780	88.88%	0.8892	89.44%	88.42%	96.52%	0.9850	0.8842	0.8803	0.8994
3	Adam	32	0.025	20%	0.2059	93.12%	0.9316	93.24%	93.10%	97.75%	0.9909	0.9310	0.9360	0.9444
4	Adam	32	0.025	20%	0.1699	94.15%	0.9418	94.47%	93.90%	98.17%	0.9939	0.9390	0.9326	0.9433
5	Adam	40	0.025	20%	0.1943	94.22%	0.9424	94.30%	94.17%	98.10%	0.9916	0.9417	0.9479	0.9540
6	Adam	40	0.025	20%	0.1318	95.46%	0.9551	95.62%	95.40%	98.54%	0.9964	0.9540	0.9515	0.9588
7	NAdam	96	0.075	50%	0.1089	95.87%	0.9587	96.00%	95.73%	98.67%	0.9971	0.9573	0.9493	0.9585
8	Adam	32	0.025	20%	0.1553	96.03%	0.9611	96.20%	96.02%	98.74%	0.9932	0.9602	0.9656	0.9696
9	NAdam	80	0.075	50%	0.1087	96.30%	0.9629	96.53%	96.05%	98.85%	0.9963	0.9605	0.9565	0.9639
10	Adam	32	0.025	20%	0.1158	96.49%	0.9650	96.66%	96.34%	98.89%	0.9956	0.9634	0.9578	0.9646
11	Adam	40	0.025	20%	0.1053	96.66%	0.9659	96.70%	96.49%	98.90%	0.9960	0.9649	0.9679	0.9725
12	NAdam	96	0.075	50%	0.0935	96.84%	0.9687	96.98%	96.77%	99.00%	0.9972	0.9677	0.9629	0.9692
13	Adam	40	0.025	20%	0.0978	96.84%	0.9688	96.95%	96.82%	98.99%	0.9970	0.9682	0.9625	0.9689
14	NAdam	72	0.050	35%	0.0970	96.92%	0.9688	96.96%	96.80%	98.99%	0.9969	0.9680	0.9649	0.9706
15	NAdam	96	0.075	50%	0.1008	96.92%	0.9694	97.02%	96.86%	99.01%	0.9967	0.9686	0.9663	0.9713

**Note:**

* PO, parameters optimizer; BS, batch size; DR, dropout ratio; MLR, model learn ratio; AUC, area under curve; IoU, intersection over union.

**Table 29:** ResNet152V2 model correlation results.

Metric	Batch size	Dropout ratio	Model learn ratio
Loss	−0.4200	0.5295	−0.0916
Accuracy	0.3011	−0.5775	−0.0420
F1	0.2995	−0.5862	−0.0447
Precision	0.3077	−0.5748	−0.0309
Recall	0.2914	−0.5960	−0.0575
Specificity	0.3100	−0.5705	−0.0272
AUC	0.3840	−0.5760	0.0391
Sensitivity	0.2914	−0.5960	−0.0575
IoU Coef	0.2161	−0.5871	−0.1328
Dice Coef	0.2380	−0.5881	−0.1108

### Top-1 promising results

**Figure 9:**
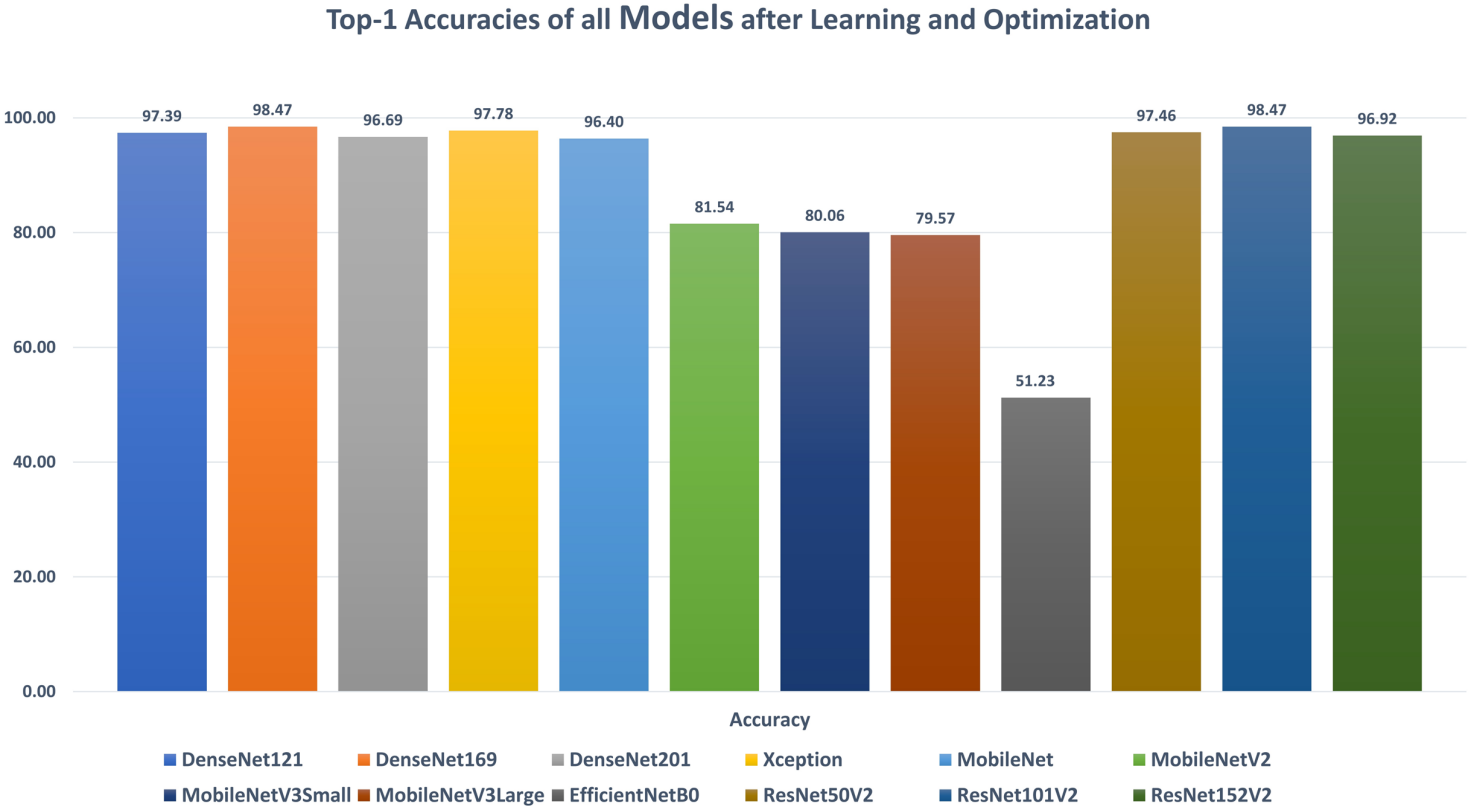
The top-1 accuracies of all reported experiments.

Table 30 reports Top-1 results of all reported experiments. They are sorted according to the sequence of the reported experiments. The best-achieved loss value was 0.0523, which the DenseNet169 model reported. The best-achieved accuracy value was 98.47% that was reported by the DenseNet169 and ResNet101V2 models. The best achieved F1 value was 0.9849, which the DenseNet169 model reported. The best-achieved precision value was 98.50% that was reported by the DenseNet169 model. The best-achieved recall value was 98.47% that was reported by the DenseNet169 model. The best-achieved specificity value was 99.50% that the DenseNet169 model reported. The best-achieved AUC value was 0.9984, which the ResNet50V2 model reported. The best-achieved sensitivity value was 0.9847, which the DenseNet169 model reported. The best-achieved IoU value was 0.9862, which the ResNet101V2 model reported. The best-achieved dice value was 0.9879, which the DenseNet169 model reported. Figure 9 summarizes the top-1 accuracies.

**Table 30:** The top-1 results of all reported experiments.

Model	PO*	BS*	DR*	MLR*	Loss	Acc.*	F1	Prec.*	Recall	Spec.*	AUC*	Sen.*	IoU*	Dice
DenseNet121	AdaMax	40	0.075	55%	0.1268	97.39%	0.9738	97.44%	97.33%	99.15%	0.9962	0.9733	0.9750	0.9783
DenseNet169	SGD	88	0.025	90%	0.0523	98.47%	0.9849	98.50%	98.47%	99.50%	0.9983	0.9847	0.9860	0.9879
DenseNet201	SGD	80	0.000	45%	0.1153	96.69%	0.9675	96.90%	96.61%	98.97%	0.9958	0.9661	0.9667	0.9711
Xception	RMSProp	80	0.525	70%	0.0888	97.78%	0.9783	97.90%	97.76%	99.30%	0.9961	0.9776	0.9750	0.9789
MobileNet	NAdam	56	0.050	25%	0.1247	96.40%	0.9636	96.39%	96.33%	98.80%	0.9952	0.9633	0.9664	0.9706
MobileNetV2	RMSProp	16	0.425	55%	2.5140	81.54%	0.8158	81.69%	81.48%	93.91%	0.9352	0.8148	0.8635	0.8728
MobileNetV3Small	RMSProp	8	0.075	100%	27.860	80.06%	0.7995	80.50%	79.43%	93.59%	0.9270	0.7943	0.8159	0.8365
MobileNetV3Large	AdaMax	16	0.175	20%	0.5192	79.57%	0.7851	83.15%	74.50%	94.97%	0.9531	0.7450	0.7550	0.7931
EfficientNetB0	RMSProp	64	0.375	80%	1.3980	51.23%	0.5070	52.92%	48.72%	85.55%	0.8003	0.4872	0.6478	0.6724
ResNet50V2	AdaGrad	16	0.100	30%	0.0792	97.46%	0.9737	97.60%	97.14%	99.20%	0.9984	0.9714	0.9635	0.9703
ResNet101V2	Adam	56	0.075	30%	0.0673	98.47%	0.9845	98.45%	98.45%	99.48%	0.9972	0.9845	0.9862	0.9878
ResNet152V2	NAdam	96	0.075	50%	0.1008	96.92%	0.9694	97.02%	96.86%	99.01%	0.9967	0.9686	0.9663	0.9713

**Note:**

* PO, parameters optimizer; BS, batch size; DR, dropout ratio; MLR, model learn ratio; Acc., accuracy; Prec., precision; Spec., specificity; AUC, area under curve; Sen., sensitivity; IoU, intersection over union.

#### Comparative study

**Figure 10:**
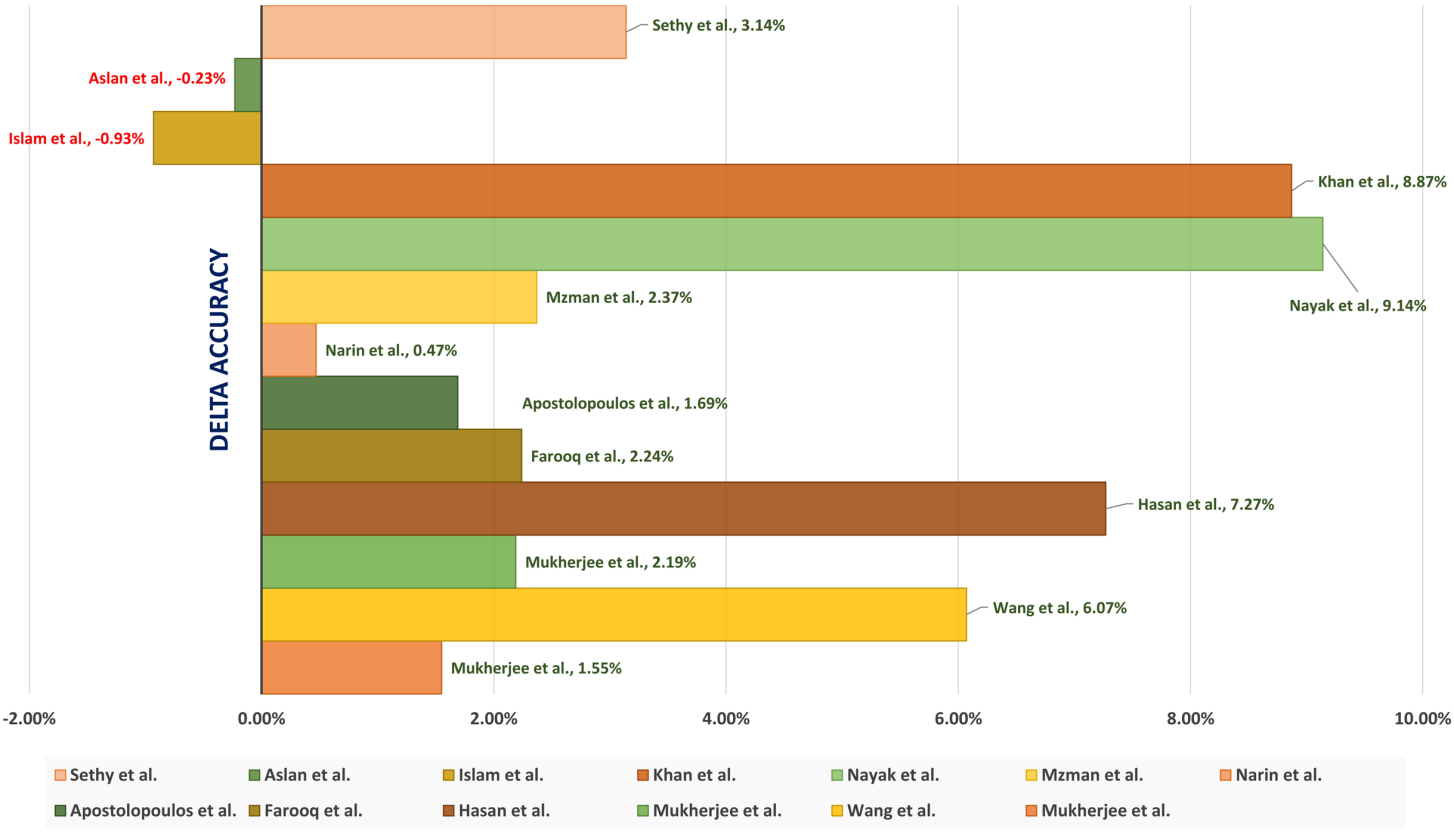
A delta comparison between the current study and related works.

Table 31 shows a comparison between the results of both the proposed OTLD-COVID-19 technique and the other state-of-the-art techniques. The techniques are ordered according to the year of publication. The recorded results show that the proposed OTLD-COVID-19 technique outperforms the four classes’ state-of-the-art compared techniques. Figure 10 depicts a delta comparison between the current study and related works concerning the accuracy. It shows that the current study accuracy exceeds 11 related works accuracies.

**Table 31:** Comparison the proposed technique with other state-of-the-art techniques.

Research	Classes #	Accuracy	Sensitivity	Specificity	Precision	F1-Score	AUC	Recall
Mukherjee et al. (2020)	2	96.92%	94.20%	100.0%	100.0%	97.01%	99.22%	N/A
Wang, Lin & Wong (2020)	3	92.40%	80.00%	N/A	88.90%	N/A	N/A	N./A
Majeed et al. (2020)	4	N/A	94.52%	99.35%	N/A	N/A	N/A	N/A
Hammoudi et al. (2020)	2	96.28%	97.90%	N/A	94.80%	N/A	N/A	N/A
Hussain et al. (2021)	4	91.20%	91.76%	93.48%	92.04%	90.04%	N/A	91.90%
Farooq & Hafeez (2020)	4	96.23%	100.0%	100.0%	N/A	100.0%	N/A	N/A
Apostolopoulos & Mpesiana (2020)	3	96.78%	98.66%	96.46%	N/A	N/A	N/A	N/A
Narin, Kaya & Pamuk (2020)	2	98.00%	96.00%	100.0%	100.0%	98.00%	100.0%	N/A
Islam et al. (2020)	2	96.10%	N/A	N/A	N/A	N/A	N/A	N/A
Nayak et al. (2021)	2	89.33%	100.0%	N/A	N/A	N/A	N/A	N/A
Khan, Shah & Bhat (2020)	4	89.60%	N/A	97.90%	93.17%	95.61%	N/A	98.25%
Islam, Islam & Asraf (2020)	3	99.40%	99.30%	99.20%	N/A	98.90%	99.90%	N/A
Aslan et al. (2021)	3	98.70%	N/A	99.30%	N/A	98.80%	99.00%	98.80%
Sethy et al. (2020)	3	95.33%	95.33%	N/A	N/A	95.34%	N/A	N/A
OTLD-COVID-19 Approach	4	98.47%	98.47%	99.50%	98.50%	98.49%	99.83%	98.47%

## Conclusion

Early detection of COVID-19 positive cases is essential to prevent the spread of this pandemic and treat affected patients quickly. This study presented an Optimized Transfer Learning-based Approach for Automatic Detection of COVID-19 (OTLD-COVID-19) approach, which adopted the Manta Ray Foraging Optimization (MRFO) algorithm to optimize the parameters and hyperparameters of twelve off-the-shelf CNN architectures. The proposed OTLD-COVID-19 approach aims to aid the radiologists in automating the classification of COVID-19 cases based on chest X-ray images. The OTLD-COVID-19 approach is built upon five essential phases. A four-class real dataset has been constructed from eight public datasets to get a relatively large number of chest X-ray images (=12,933 images). Besides, data augmentation is performed to increase the size of the training set and enhance generalization. The training and testing ratio of the dataset was set as 85% and 15%, respectively. The obtained experimental results showed that the proposed OTLD-COVID-19 approach achieved high-performance metrics that outperformed the compared approaches. To extend this work, Generative Adversarial Networks (GAN) can supplement the lack of training set to improve the performance of the classification process. In expansion, the number of other diseases causing pneumonia may be expanded, and the proposed approach can be utilized to distinguish them from the COVID-19. This study could also be extended to other diseases to help the healthcare system respond more effectively during any possible future pandemic.

## Additional Information and Declarations

### Data Availability

The following information was supplied regarding data availability: The source code is available at GitHub: https://github.com/HossamBalaha/An-Optimized-TL-based-Approach-for-Auto.-Detection-of-COVID-19-from-Chest-X-ray-Images. The third-party data is available at: 1. COVID-19 Radiography Database. https://github.com/ieee8023/covid-chestxray-dataset. 2. Pneumonia (virus) vs COVID-19. https://www.kaggle.com/muhammadmasdar/pneumonia-virus-vs-covid19. 3. Covid-19 Xray images using Cnn. https://www.kaggle.com/akkinasrikar/covid19-xray-images-using-cnn. 4. COVID-19 X-ray Images5. https://www.kaggle.com/uddiptadas/covid19-xray-images5. 5. COVID-19 Patients Lungs X Ray Images 10000. https://www.kaggle.com/nabeelsajid917/covid-19-x-ray-10000-images. 6. COVID-19 Chest X Rays. https://www.kaggle.com/rupeshs/covid19-chest-x-rays. 7. COVID-19 Dataset. https://www.kaggle.com/syedrz/covid19-dataset. 8. Curated Chest X-Ray Image Dataset for COVID-19. https://www.kaggle.com/unaissait/curated-chest-xray-image-dataset-for-covid19.
